# Role of Breastfeeding and Complementary Food on Hemoglobin and Ferritin Levels in a Cambodian Cross-Sectional Sample of Children Aged 3 to 24 Months

**DOI:** 10.1371/journal.pone.0150750

**Published:** 2016-03-14

**Authors:** Anika Reinbott, Irmgard Jordan, Johannes Herrmann, Judith Kuchenbecker, Ou Kevanna, Michael B. Krawinkel

**Affiliations:** 1 Justus Liebig University, Wilhelmstr. 20, 35392 Giessen, Germany; 2 National Maternal and Child Health Center, Phnom Penh, No. 31A, Rue de France (St. 47), 12202 Phnom Penh, France, Phnom Penh, Cambodia; University of São Paulo, BRAZIL

## Abstract

**Background:**

Iron deficiency derives from a low intake of dietary iron, poor absorption of iron, and high requirements due to growth as well as blood loss. An estimated number of about 50% of all anemia may be attributed to iron deficiency among young children in Cambodia.

**Methods:**

A cross-sectional survey was conducted in rural Cambodia in September 2012. Villages in pre-selected communes were randomly chosen using stunting as a primary indicator of nutritional status. In total, 928 randomly selected households with children aged 3–23 months were included. Hemoglobin, ferritin, soluble transferrin receptor (sTfR), and retinol binding protein (RBP) were assessed from capillary blood samples. In addition, length/height and weight of mothers and children were taken and data on dietary diversity was collected. A child feeding index (CFI) was created. Associations between biomarkers of iron and vitamin A status and nutritional status or food intake were explored.

**Results:**

Anemia prevalence was highest among 6- to 12-months-olds (71%). Ferritin and sTfR inversely correlated and were significantly associated with hemoglobin concentrations. The consumption of animal source foods (ASF) significantly impacts on the interaction between ferritin, sTfR and hemoglobin. Concentrations of RBP were significantly higher in children who had received a vitamin A supplement. The CFI was associated with sTfR and hemoglobin. Lower length and weight were associated with lower ferritin levels and showed an indirect effect on hemoglobin through ferritin.

**Conclusion:**

Nutrition programs targeting children under 2 years of age need to focus on the preparation of complementary foods with high nutrient density to sustainably prevent micronutrient deficiency and generally improve nutritional status. Future assessments of the micronutrient status should include identification of hemoglobinopathies and parasitic infections to better understand all causes of anemia in Cambodian infants and young children.

**Trial Registration:**

German Clinical Trials Register DRKS00004379

## Introduction

Iron deficiency is estimated to be globally the most common cause of anemia. [1] The World Health Organization (WHO) estimates that worldwide about 50% of all anemia can be attributed to iron deficiency which derives from an imbalance between intake and needs, with factors such as low bioavailability of dietary iron and high requirements due to growth and blood loss. [1] Pregnant women and young children are especially vulnerable to iron deficiency. [1–4] Body iron stores decline rapidly during the first 6 months of life, and after that many infants are at risk of iron deficiency. [5] Children suffering from iron deficiency anemia in infancy may have long-term developmental disadvantages reflected in impaired motor and mental development, and growth. [6] Exclusive breastfeeding of children younger than 6 months of age is known to protect the infant’s body from infections that increase the risk of iron deficiency. [7] The best sources of dietary iron for children of complementary feeding age are animal source foods (ASF). Due to low affordability and poor nutritional knowledge, however, ASF are generally lacking in children’s diets in developing countries. [8] Biomarkers often used to assess iron deficiency are serum ferritin and soluble transferrin receptor (sTfR). Ferritin, a positive acute response protein, is known to be normally high at birth. [9] Ferritin levels rise during the first two months of age and then fall until the end of the first year of life, i.e. later infancy. At about one year of age ferritin levels begin to rise again. [10] At this age, serum ferritin levels below 12 μg/L indicate a depletion of iron stores. Concentrations of sTfR increase when serum ferritin levels fall. [11] STfR levels above 8.3 mg/L indicate tissue iron deficiency. Both, ferritin and sTfR levels are known to increase during inflammation episodes.

Besides a lack of dietary iron, other causes of anemia in infancy can be deficiencies of vitamin B12 or vitamin A, hemoglobinopathies, parasite infestations, and chronic infections. However, the availability of data on the two latter causes is low, especially for infants below 6 months of age in Cambodia. Vitamin A status is often measured as concentrations of plasma retinol binding protein (RBP). Its linkage to iron metabolism still lacks evidence, while vitamin A is likely to play a role in the release of iron from the liver. [12]

The most recent Demographic and Health Survey in Cambodia (CDHS 2010) indicated that anemia is a severe public health problem: the highest prevalence of anemia was found among children under 2 years of age, ranging from 81% among 6- to 8-month-olds to 70% (N = 190) among 18- to 23-month-olds (N = 415). [13] Children under 5 years of age in the lowest wealth quintile were more likely to be anemic than children in the highest quintile (60% and 43%, respectively). The most common type of anemia was moderate anemia with hemoglobin levels between 70–99 g/L. [13] No national data on hemoglobinopathies are available, but several studies suggest that hemoglobin E is the most prevalent form of hemoglobinopathy, with 30–60% of the population being a carrier. [14]

The objective of this study was to explore relationships between biomarkers of iron status in children aged 3–23 months and their linkage to hemoglobin levels by considering age and sex of the child, maternal hemoglobin status, age appropriate feeding practices and the food intake of either breastmilk or ASF. Furthermore, associations of the biomarkers with anthropometric data were analyzed.

## Methods

### Study Sites and Study Population

The survey was carried out with data collected for a cross-sectional nutrition baseline survey conducted from 10 September to 07 October, 2012, in collaboration with a food security project of the Food and Agriculture Organization (FAO) of the United Nations in Preah Vihear and Otdar Meanchey provinces of Cambodia. In total, 16 communes from six districts were selected. [15] About 17,650 possible beneficiaries of the FAO project consisting of rural farming households lived in this area at the time of the survey. Only households with children aged 0–23 months were eligible to participate in the survey. Other inclusion criteria were: residency in the sampled area, random selection, and willingness to participate in the survey. (S1 Fig)

### Ethical Considerations

The study was approved by the Institutional Review Board of the Faculty of Medicine at Justus Liebig University, Giessen, Germany (26 July 2012) and the National Ethics Committee for Health Research in Phnom Penh, Cambodia (17 August 2012); it was registered in the German Clinical Trials Register (no. DRKS00004379) only within the first week of recruiting participants. Internet connection problems caused the short delay. Prior to collecting data and blood samples a written informed consent was obtained from all mothers and caregivers by signature or fingerprint. The ethical committees approved the consent format prior to data collection. The authors confirm that all ongoing and related trials for this intervention are registered. ()

### Design

Using the *Emergency Nutrition Assessment (ENA) for Smart* sample size calculator and considering a population size of 15,000 children under two years of age in the surveyed area, 50% of stunting (primary indicator), a desired precision of +/- 5%, and a design effect of 3, the sample size calculation resulted in 1,124 children. [16] A large design effect of three was chosen as no estimation of intra class correlation coefficient (ICC) was available prior to the data collection. The sampling was conducted using a two-stage probability sampling strategy with communes as clusters. Initially three villages per commune were sampled proportional to population size. For the second sampling stage, 23 households with children aged 0–23 months per village were randomly selected. (S1 text)

### Data Collection

Semi-structured questionnaires were administered in face-to-face interviews with the primary caregiver of the child below two years of age in the selected household and included a household, child, and caregiver section. The data assessment included socio-economic and demographic data, household and child dietary diversity scores based on a 24-hour recall and 7-day food frequency, child’s health data, as well as feeding and caring practices. [17,18] Anthropometric measurements were taken with standardized equipment from Seca (Seca GmbH & Co KG, Hamburg, Germany; d = 0.05). Mothers’ weight and height were collected as well as the weight and length of the child following the FANTA protocol. [19] The maximum tolerated difference between the first and second measurement was 1.0 cm for height/length and 0.5 kg for weight. Height/length and weight were assessed to the nearest 0.1 cm and 0.1 kg, respectively. All measurements were taken twice and the mean was used for further analysis. All data collection tools were pre-tested in the field.

Child’s nutritional status was estimated by calculating length-for-age Z-scores (LAZ), weight-for-age Z-scores (WAZ), and weight-for-length Z-scores (WLZ) finally following the WHO protocol. [20]

### Blood data collection and analysis

One capillary blood sample was taken to assess hemoglobin status of both mothers and children using a HemoCue^®^ device (HemoCue^®^ 301, Ängelholm, Sweden). Additional capillary blood samples of 100 to 150 μl were collected from children aged ≥3 months. Blood was drawn using a Microvette^®^ (Saarstedt 20.1292), which was sealed and afterwards inverted 8–10 times. The sample was labelled and stored on ice. No longer than 12 hours after the first sample was collected, the samples were centrifuged (for 5 minutes at 2,000*g) using a Mini-Centrifuge at 2,000 relative centrifugal force (RCF) (g force) for 5 minutes in the Microvettes^®^ in order to obtain plasma. The plasma was pipetted into labelled 0.2 ml Multiply^®^ (PCR tubes). Due to the lack of access to a freezer in the research field, plasma samples were then either stored cool for a maximum of 7 days and then put into a freezer or stored in a freezer on the same day following the guidelines from Erhardt. [21] In the guidelines a reference is made to extensive tests at the Center of Disease Control and Prevention (CDC) in Atlanta which have shown that proteins in serum are stable at room temperature for one week.

The samples were analyzed in a laboratory (VitMin Lab, Willstaett, Germany) where serum ferritin, soluble transferrin receptor (sTfR), retinol binding protein (RBP), acidic glycoprotein (AGP), and C-reactive protein (CRP) levels indicating subclinical infection or inflammation were analyzed using a Sandwich Enzyme-Linked Immunosorbent Assay (ELISA). [22,23]

CRP and AGP were used for classifying the inflammation stage of a child: no inflammation (CRP levels ≤5 mg/L and AGP levels ≤1 g/L), inflammation stage (IS) I (CRP levels >5 mg/L and AGP levels ≤1 g/L), IS II (CRP levels >5 mg/L and AGP levels > 1 g/L), IS III (CRP levels ≤ 5 mg/L and AGP levels >1 g/L). [24]

Prevalence of anemia was calculated for children aged 3–23 months according to WHO guidelines. [25] Children were considered anemic when hemoglobin concentrations were <110 g/L (mild anemia: 100–109 g/L, moderate anemia: 70–99 g/L, severe anemia: <70 g/L). Anemic non-pregnant mothers had a hemoglobin concentration below 120 g/L (mild anemia: 100–119 g/L, moderate anemia: 70–99 g/L, severe anaemia: <70 g/L). For children aged 3–6 months additional cut-offs suggested by Domellöf et al were used: hemoglobin <105 g/L. [26]

Ferritin and sTfR were adjusted for inflammation following the recommendation by Thurnham et al. and Grant et al., respectively [24,27], where children aged ≥6 months were considered iron deficient when ferritin levels were <12.0 μg/L (iron storage depletion). Tissue iron deficiency was defined as sTfR levels ≥8.3 mg/L for children aged 3–23 months. [24,27] Iron deficiency anemia (IDA) was defined as anemia concurrent with iron deficiency with either low ferritin levels, low sTfR levels, or both.

Vitamin A deficient children had RBP levels <0.7 μmol/L. [28] RBP was only analyzed for children without any signs of inflammation. Different cut-offs and adjustments for inflammation status are summarized in Table 1.

**Table 1:** Cut-offs and correction factors for hemoglobin, ferritin, and sTfR [2,4,2,7].

		Correction factor for inflammation^1^				
	Cut-off	Age gro up	Stage I	Stage II	Stage III	Reference
hemoglobin (g/L)	<110	6–36 months	0.99	1.12	1.03	Grant et al. 2012
ferritin (μg/L)	<12	<5 years	0.77	0.53	0.75	Thurnham et al. 2010
sTfR (mg/L)	≥8.3	6–36 months	1.03	0.87	0.96	Grant et al. 2012

sTfR = soluble transferrin receptor

^1^ all references use a cut off of >5 mg/L for CRP (C-reactive protein) and >1 g/L for AGP (α-1 acid glycoprotein) as defined by Thurnham et al. 2003. [24]

### Indicators for infant and young child feeding

Feeding practices were assessed using the WHO IYCF indicator for exclusive breastfeeding for children ≤6 months of age. [18,29] The consumption of iron-rich foods within the past 24 hours was reduced to ASF only, indicating that the child aged 6–23 months had received either organ meat, flesh meat, eggs, or fish. The consumption of milk has been excluded, as dairy products are not commonly consumed in the region. Furthermore, a child feeding index (CFI) [15] for children aged 6–23 months was calculated consisting of five different components: still being breastfed, not bottle-fed, dietary diversity, meal frequency, and food frequency. The CFI score ranged from 0–10, with 10 being the best score.

### Statistical analysis

All data were entered twice into EpiData (version 3.1) [30] and analyzed with SPSS (IBM, SPSS Statistics version 22.0.0.1) and Stata (Stata Corp, USA, version 10.1). Ferritin and sTfR were adjusted for inflammation and log-transformed throughout the analyses. RBP was analyzed only for children without any signs of inflammation. Distributions of hemoglobin, ferritin, sTfR, and RBP values were initially examined via graphic analysis. No considerable clustering effects were seen in the sample. Chi^2^-tests were used to determine associations between dichotomous variables and odds ratio was calculated and used as effect size. Independent sample t-tests were applied and the effect size r [31] was calculated. Moderator and mediator models were applied using the PROCESS module of Andrew Hayes [32], to assess the role of different moderators and mediators on the relationship between different biomarkers of iron status and hemoglobin levels. A mediator variable can be understood as a potential causal link between independent and dependent variable. In a moderator model, the relationship between an independent and dependent variable depends on the level of the moderator, the third variable. Kappa-squared and bootstrap confidence intervals were calculated. For moderator models grand mean centering was applied for the predictors age, ferritin, and sTfR. Sex of the child was not included as a covariate since it did not show any significant effect. Age and age^2^ were included as covariates in moderator models in order to account for linear and/or curvilinear distributions.

## Results

A total of 928 complete datasets including anthropometric and blood data as well as all other information were available for the analyses (S2 text).) The main child characteristics are reported in Table 2 disaggregated into three age groups: 3<6 months, 6<12 months, and 12<24 months. Overall, prevalence of inflammation was low. Mean LAZ, WLZ, and WAZ scores (±SD) were lowest in the oldest age group (LAZ: -1.50 (0.99), WLZ: -0.99 (0.93), WAZ: -1.46 (0.94)). Continued breastfeeding was practiced by most caregivers resulting in 71% of the 12- to 24-month-olds still being breastfed. The consumption of ASF in the past 24 hours was higher among 12- to 24-month-old children (91%) than among the 6- to 12-month-olds (66%). Vitamin A supplement had been received by 81% (N = 920) of children aged 3–24 months six months prior the survey with the highest coverage among the 3- to 6-month-olds (97%). Deworming tablets had been received less often (38%, N = 912) with the 18- to 24-months-olds showing the highest coverage (52.6%). Thirty-five percent of all children (N = 902) received both, vitamin A capsules and deworming tablets. The number of children aged 6<24 months who received a vitamin A supplement, a deworming tablet in the past 6 months, and had consumed ASF in the past 24 hours was low (31%, N = 783). The highest child feeding index scores were given to the 6- to 12-month-olds (mean (±SD) = 7.73 (1.75)).

**Table 2:** Main child characteristics: anthropometry, IYCF, and micronutrient status.

	3<6 months	6<12 months	12<24 months
**N**	125	332	471
% inflammation*	11.5	25.4	23.2
**Anthropometry**			
**Mean (±SD) length-for-age Z-score**	-0.95 (0.89)	0.90 (1.24)	-1.50 (0.99)
**Mean (±SD) weight-for-length Z-score**	0.03 (1.02)	-0.45 (1.07)	-0.99 (0.93)
**Mean (±SD) weight-for-age Z-score**	-0.66 (0.99)	—0.87 (1.08)	-1.46 (0.94)
**Nutrition**			
**% Exclusively breastfed**	75.2	n/a	n/a
**% Still breastfed**	96.0	97.0	71.3
**% Consumed ASF in past 24 hours**	n/a	66.3	91.1
**Mean (±SD) child feeding index (children aged 6–23 months)**	n/a	7.73 (1.75)	5.96 (1.70)
**% Deworming tablet in past 6 months**	12.1	26.8	52.6
**% Vitamin A supplement in past 6 months**	96.9	80.6	84.4
Anemia status and prevalence			
**Mean (±SD) Hb (g/L)**	109.16 (11.68)	103.49 (11.71)	107.44 (13.51)
**% Anemia (Hb < 105 g/L)**	26.8	n/a	n/a
**% Anemia (Hb < 110 g/L)**	50.0	70.9	51.1
**% Mild Anemia (Hb 100<109.9 g/L)**	33.6	35.9	24.0
**% Moderate Anemia (Hb 70<99.9 g/L)**	16.4	34.3	26.8
**% Severe Anemia (Hb <70 g/L)**	0.0	0.6	0.2
**Iron status and prevalence**			
**Mean (95% CI) Ferritin (μg/L)**	50.6 (42.7, 60.1)	15.4 (13.8, 17.1)	14.3 (13.2, 15.4)
**Mean (95% CI) sTfR (mg/L)**	7.7 (7.3, 8.1)	10.3 (9.9, 10.8)	10.1 (9.7, 10.5)
**Prevalence of Iron Deficiency (ID)**			
**% Ferritin <12 μg/L**	6.6	42.9	42.5
**% sTfR >8.3 mg/L**	28.7	65.3	61.1
**Prevalence of Iron Deficiency Anemia (IDA)**			
**% Hb <110g/L and Ferritin <12μg/L**	0.0	3.8	4.2
**% Hb <110g/L and sTfR >8.3 mg/L**	23.0	26.9	25.0
**% Hb <110g/L, Ferritin <12μg/L & sTfR >8.3 mg/L**	11.5	43.6	54.6
**Vitamin A status**			
**Mean (±SD) RBP (μmol/L)**	1.05 (1.00, 1.10)	1.06 (1.03, 1.09)	1.08 (1.05, 1.10)
**% RBP**^1^ **<0.7 μmol/L**	1.9	2.8	1.1
**Maternal Hemoglobin**			
**Mean (±SD) Hb (g/L)**	125.60 (11.53)	125.64 (12.23)	126.70 (13.37)
**% Anemia**^2^ **(Hb <110 g/L)**	30.4	30.8	27.3
**Household characteristics**			
**Mean (±SD) household dietary diversity score**	6.54 (1.59)	6.85 (1.74)	6.74 (1.70)
**Mean years of caregiver’s schooling**	4.06 (3.60)	3.69 (3.25)	3.45 (3.15)
**% Protected source of drinking water**	84.0%	86.4%	86.6%
**% Improved sanitation facilities**	20.0%	19.6%	17.4%

ASF = animal source foods; Hb = hemoglobin; sTfR = soluble transferrin receptor; RBP = retinol binding protein; SD = standard deviation; n/a = not applicable

*All biomarkers were adjusted for inflammation as stated in Table 1; inflammation was defined following Thurnham et al. 2003: >5 mg/L for CRP (C-reactive protein) and >1 g/L for AGP (α-1 acid glycoprotein) (see Table 1)

^1^only children without any signs of inflammation;

^2^only non-pregnant mothers

**Fig 1:**
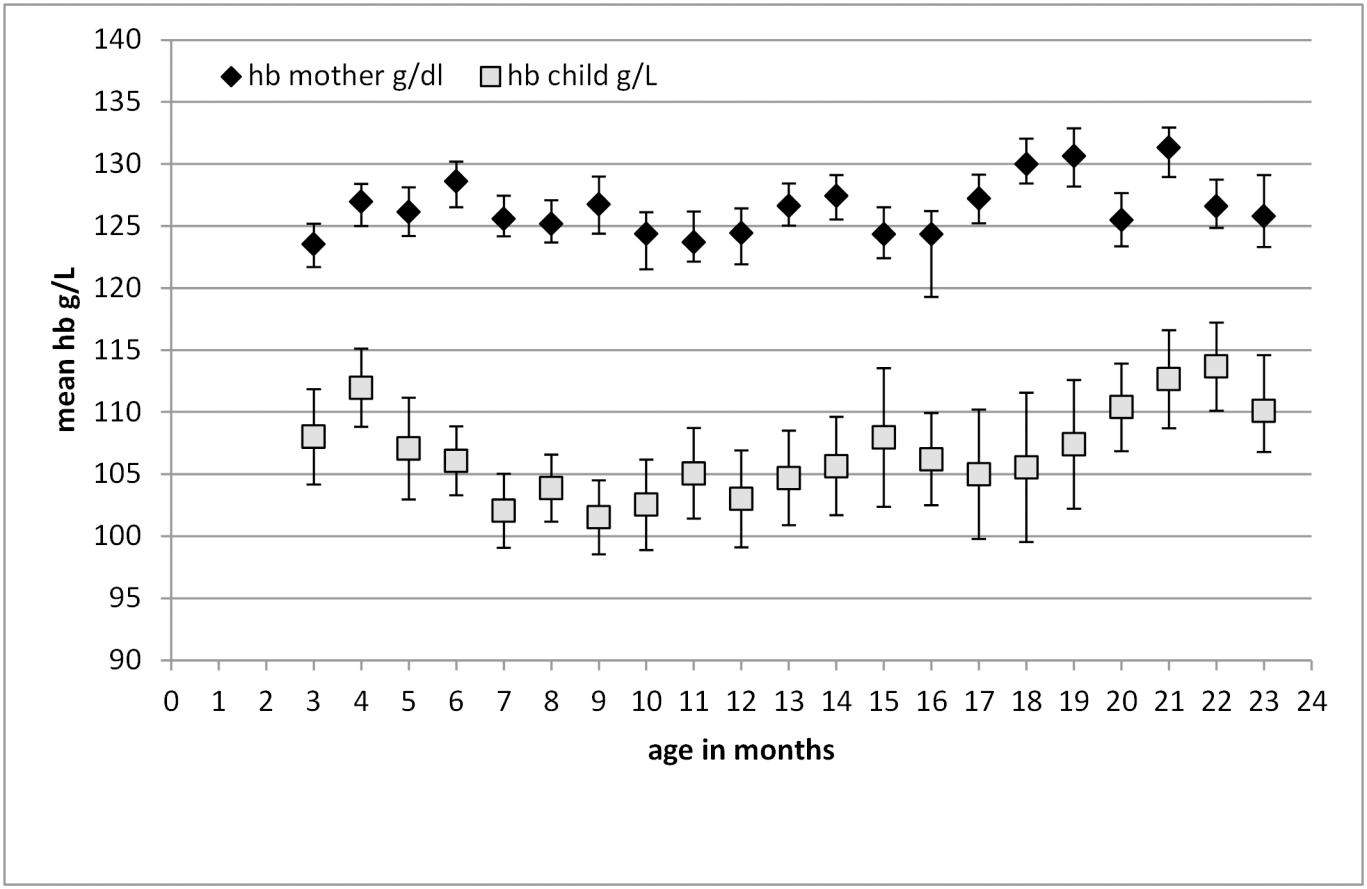
Mean child hemoglobin concentrations (±SD) by age groups in months with respective maternal hemoglobin concentrations. 3<4 mo: N = 38, 4<5 mo: N = 45, 5<6 mo: N = 39, 6<7 mo: N = 52, 7<8 mo: N = 55, 8<9 mo: N = 55, 9<10 mo: N = 56, 10<11 mo: N = 57, 11<12 mo: N = 55, 12<13 mo: N = 60, 13<14 mo: N = 53, 14<15 mo: N = 44, 15<16 mo: N = 28, 16<17 mo: N = 46, 17<18 mo: N = 32, 18<19 mo: N = 29, 19<20 mo: N = 25, 20<21 mo: N = 37, 21<22 mo: N = 42, 22<23 mo: N = 40, 23<24 mo: N = 33. mo = months.

**Fig 2:**
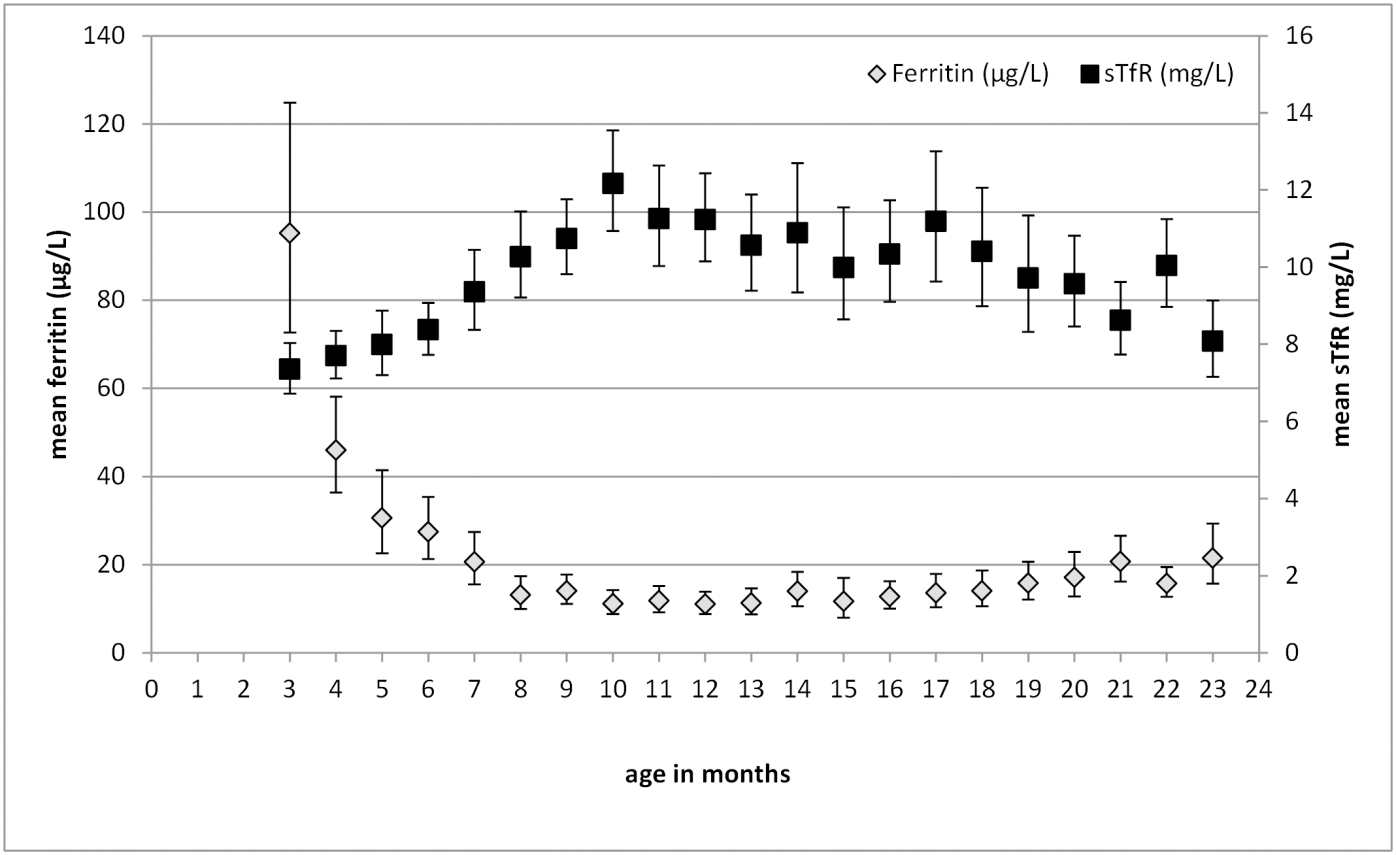
Geometric means of ferritin and sTfR concentrations by age in months. 3 mo: N = 38, 4mo: N = 45, 5mo: N = 39, 6mo: N = 52, 7mo: N = 55, 8mo: N = 55, 9mo: N = 57, 10mo: N = 57, 11mo: N = 55, 12mo: N = 60, 13mo: N = 53, 14mo: N = 44, 15mo: N = 28, 16mo: N = 46, 17mo: N = 32, 18mo: N = 29, 19mo: N = 26, 20mo: N = 37, 21mo: N = 42, 22mo: N = 40, 23mo: N = 33; mo = months.

Hemoglobin levels were available from 892 biological mothers. With a mean (±SD) age of 26.7 (5.9) years, mothers had a mean (±SD) hemoglobin concentration of 126.0 (1.3) g/L. Mean values in Figs 1 and 2 are presented by age groups in completed months. Hemoglobin concentration is lowest among children aged 9 months and highest in 22-month-old children (Fig 1). Ferritin and sTfR concentrations were negatively correlated with ferritin decreasing and sTfR increasing from the youngest to the 10-month-olds (Fig 2). From 10 months of age onwards, ferritin increased slightly until 24 months, and sTfR inversely decreased. Prevalence of iron deficiency with either increased sTfR or decreased ferritin concentration was highest among children older than 12 months. IDA was found in 73.5% (N = 541) of all anemic children using the WHO cut-off. Overall, vitamin A deficiency was very low (Table 2).

Prevalence of anemia in non-pregnant mothers was 29% (N = 67) (Table 2). Maternal and child hemoglobin concentrations were weakly but significantly associated including age of the child as covariate: R^2^ = 0.022, B (SE) = 1.126 (0.337), p< 0.005 (Fig 1).

There was a significant association between maternal anemia and child anemia only in children aged 6<24 months (3<6 mo: χ² (1) = 0.97, p = 0.325; 6<12 mo: χ² (1) = 3.98, p = 0.046; 12<24 mo: χ² (1) = 7.12, p = 0.008). Based on the odds ratio, the odds for children aged 6<12 months were 1.77 times higher if their mother was anemic. Children aged 12<24 months had a 1.82 times higher chance of being anemic if their mother was anemic.

**Fig 3:**
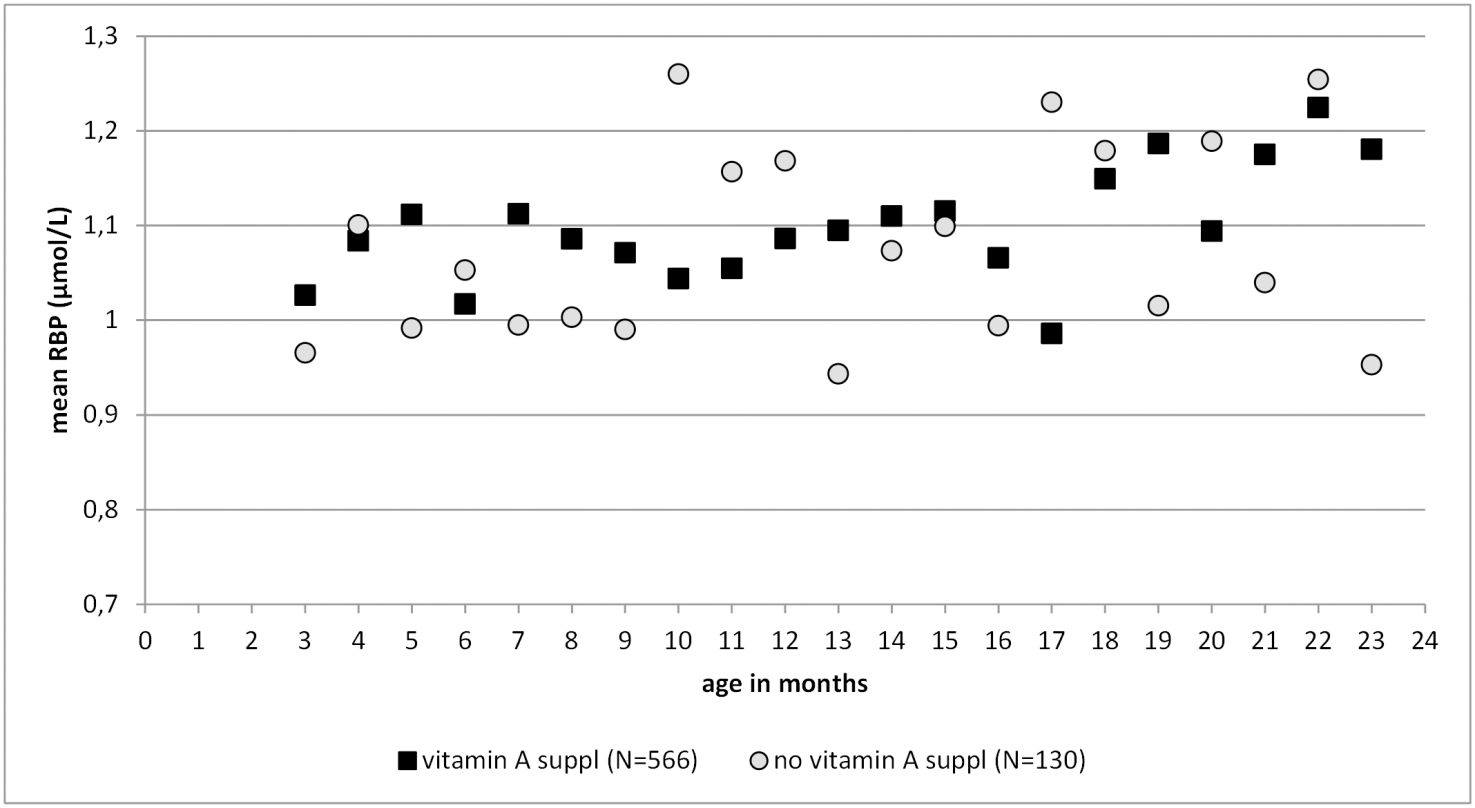
Mean RBP concentrations by age groups in months for children without any signs of inflammation only. 3 mo: N = 38, 4mo: N = 45, 5mo: N = 39, 6mo: N = 52, 7mo: N = 55, 8mo: N = 55, 9mo: N = 57, 10mo: N = 57, 11mo: N = 55, 12mo: N = 60, 13mo: N = 53, 14mo: N = 44, 15mo: N = 28, 16mo: N = 46, 17mo: N = 32, 18mo: N = 29, 19mo: N = 26, 20mo: N = 37, 21mo: N = 42, 22mo: N = 40, 23mo: N = 33; mo = months.

Mean RBP concentration increased slightly with increasing age, starting with a mean of 1.00 μmol/L in the youngest age group and ending with 1.15 μmol/L in the oldest one (Fig 3). Overall, there was a not significant difference in RBP concentrations between children who had received a vitamin A supplement in the past six months (arithmetic mean (95% CI): 1.12 (1.10, 1.14), geometric mean (95% CI): 1.09 (1.08, 1.11)) and the ones who had not received a vitamin A supplement (arithmetic mean (95% CI): 1.08 (1.03, 1.11), geometric mean (95% CI): 1.04 (1.01, 1.08)): t(df) = -1.93 (709)) p = 0.054, r = 0.07.

**Fig 4:**
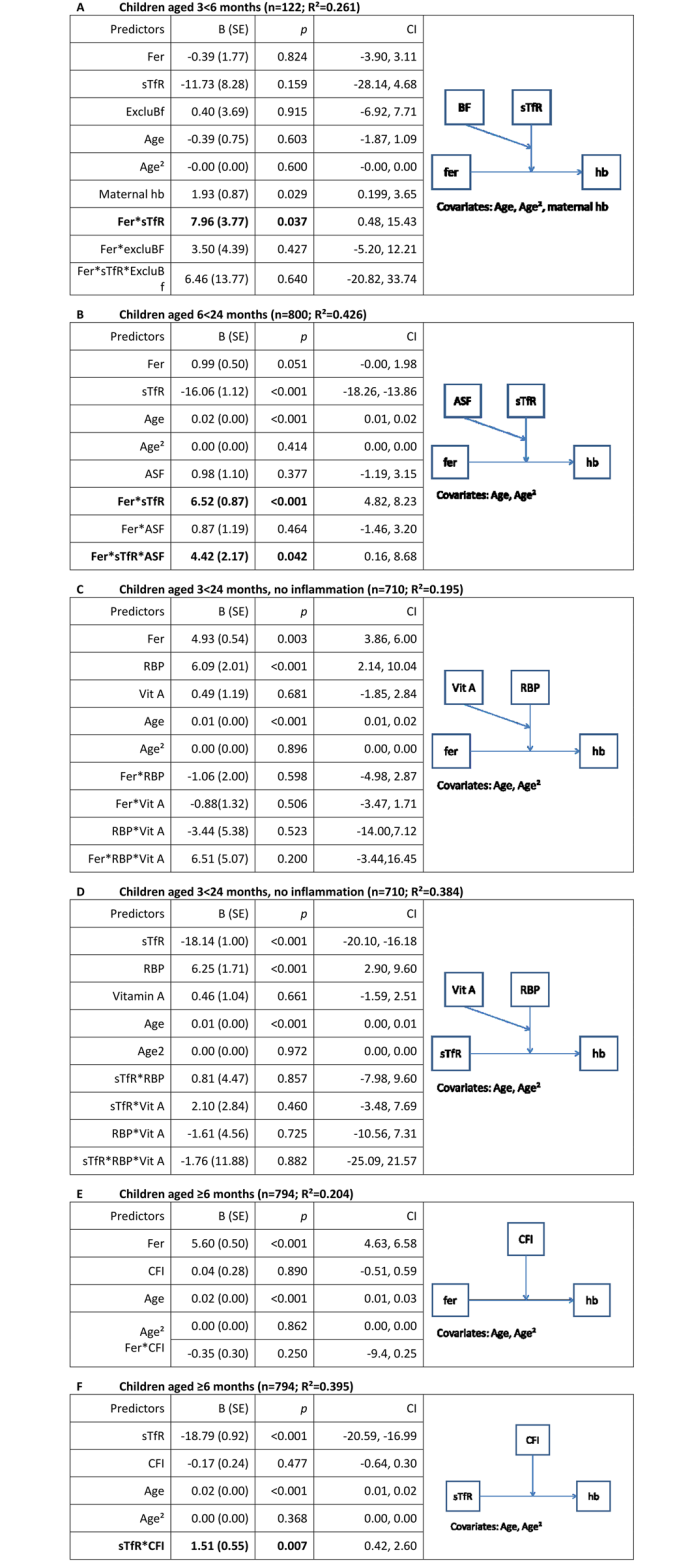
a-f. Results from moderator analyses with hemoglobin as outcome variable. Fer = Ferritin (μg/L); sTfR = soluble transferrin receptor (mg/L); ExcluBf = exclusive breastfeeding; hb = maternal hemoglobin (g/L); RBP = retinol binding protein (μmol/L); ASF = animal source foods; Vit A = vitamin A supplement; CFI = child feeding index [15]; Age = age of child (days); Fer and sTfR have been used as logarithmized (natural logarithm) continuous variables; ExcluBf and Vit A is a dichotomous variable 1 = yes, 0 = no; RBP (μmol/L) as continuous variable; CFI comprising five different indicators (breastfeeding, bottle feeding, dietary diversity, food and meal frequency) ranges from 0 to 10; R^2^ is the effect size of the entire model including covariates.

Fig 4 summarizes the main outcomes from moderator analyses where predictors of hemoglobin were centered beforehand. Ferritin, sTfR, and the interaction between ferritin and sTfR were significantly and strongly associated with hemoglobin levels for children aged 6<24 months (Fig 4b). The conditional effect of ferritin on hemoglobin at different values of sTfR as moderator showed that ferritin had a negative effect (effect -1.35, p = 0.02) on hemoglobin under the condition that sTfR is the mean of sTfR -1SD (1SD = 0.40). At mean sTfR and mean sTfR+1SD, the effect became positive: effect 0.91, p = 0.06, and effect 3.17, p<0.001, respectively.

For children aged 3<6 months the moderation model shows a significant interaction between ferritin and sTfR on hemoglobin. The higher the sTfR concentrations are, the stronger the association between ferritin and hemoglobin. The interaction in this age group is stronger and only significant for exclusively breastfed infants aged 3<6 months: B (SE)_exclu bf_ = 9.60 (2.40), *p*<0.001 vs. B (SE)_non-exclu bf_ = 3.14 (13.43), *p* = 0.816 (Table 3).

**Table 3:** Effect sizes of moderator models.

		Effect^1^	SE	*p*	LLCI	ULCI	n
**Exclusive breastfeeding (ExcluBf)**	no	3.14	13.43	0.816	-23.48	29.75	31
	yes	9.60	2.40	<0.001	4.84	14.36	94
**Animal Source Foods (ASF)**	no	2.95	1.93	0.126	-.84	6.73	154
	yes	7.37	0.98	<0.001	5.46	9.29	649

^1^effect of interaction between ferritin and sTfR on hemoglobin; LLCI = lower level for confidence interval; ULIC = upper level for confidence interval

For children aged 6<24 months, the interaction between ferritin, sTfR, and consumption of ASF was significant (B (SE) = 4.42 (2.17), p<0.05). The conditional effect of the interaction between ferritin and sTfR on hemoglobin was significantly stronger if ASF had been consumed in the past 24 hours (Table 3).

Including RBP in a model as the moderator, ferritin, sTfR, and RBP alone were significantly associated with hemoglobin (Fig 4d and 4e). The interactions, however, considering vitamin A supplementation and age as covariates, did not achieve significance.

The CFI score was not significantly associated with the relationship between ferritin and hemoglobin whereas the interaction between sTfR and CFI was significantly associated with hemoglobin levels: B(SE) = 1.51 (0.55), p<0.005. The negative relationship between sTfR and hemoglobin was stronger the lower the CFI (Fig 4f).

**Fig 5:**
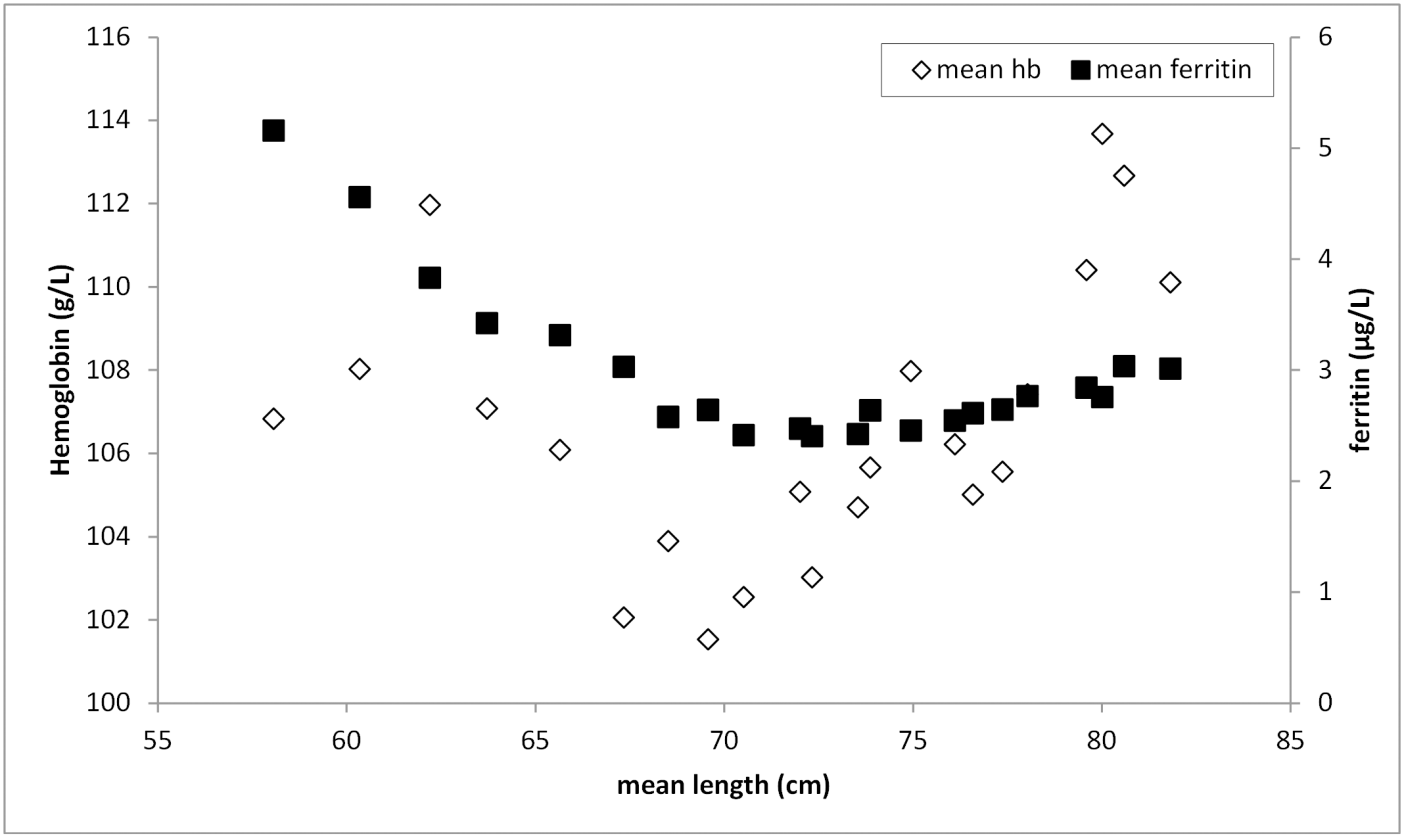
Mean hemoglobin and ferritin by mean length of each completed month age group. Hb = hemoglobin, length = mean length of each completed age groups.

There was a significant but weak direct effect of length of the child on hemoglobin concentrations (B (SE) = -0.050 (0.005), p<0.001) illustrated in Fig 5. In a mediator model, there was a significant negative indirect effect of length on hemoglobin through ferritin, b = -0.287, BCa CI [-0.362, -0.221]. This represents a medium effect, κ² = 0.156, 95% BCa CI [0.122, 0.191]. There was no significant direct effect of weight on hemoglobin, but weight had a significant negative indirect effect on hemoglobin through ferritin, b = -1.303, BCa CI [-1.641, -1.024]. The effect was of medium strength at κ² = 0.146, 95% BCa CI [0.118, 0.179]. LAZ and WLZ were also indirectly associated with hemoglobin through ferritin: b_LAZ_ = -0.086, BCa CI [-0.0336, 0.240], b_WLZ_ = 0.163, BCa CI [-0.102, 0.480]. This represents a relatively small effect for LAZ: κ² = 0.008, 95% BCa CI [0.000, 0.025]; and for WLZ: κ² = 0.015, 95% BCa CI [0.001, 0.043].

## Discussion

Assuming that infant’s iron stores are replete at birth, it is important that effective breastfeeding and the introduction of complementary food from 6 months of age onwards supply the growing body with a sufficient amount of iron. [33,34] In this study, the consumption of ASF had a significant effect on the interaction between ferritin and sTfR and their effect on hemoglobin in infants 6<24 months of age. At low levels of sTfR, the association between ferritin and hemoglobin was negative while it changed direction at higher levels of sTfR, thereby indicating iron deficiency. Consuming ASF made this association stronger when compared to findings in children who were not fed ASF.

This so-called ‘anemia of late infancy’ just reflects normal physiological changes in hemoglobin levels in the fast-growing infant. [4] In this study, neither exclusive breastfeeding, nor vitamin A supplementation showed any significant association; maternal hemoglobin showed a significant but weak association with the child’s hemoglobin status. Low hemoglobin concentrations were significantly associated with lower ferritin levels, increased sTfR levels, and lower RBP.

Iron content in breastmilk is fixed. [35] Whether it is sufficient to cover the needs of the infant is currently underexpert’s debate. For children below 6 months of age, exclusive breastfeeding is especially recommended for protecting the infant from anemia associated with parasite infestations as well as bacterial and/or viral infections, e.g. from contaminated water. [36,37] In the population studied here, exclusive breastfeeding rates were high and did not show any direct association with the biomarkers analyzed. As expected in exclusively breastfed infants the association between ferritin and hemoglobin was negative for low sTfR and positive for high sTfR. For non-exclusive breastfed infants the association between ferritin and hemoglobin was negative at all levels of sTfR. This could show that exclusive breastfeeding supports the expected correlations between biomarkers in the young body, whereas a lack of exclusive breastfeeding leads to different interactions between biomarkers of iron status. However, the interpretation of this result is difficult due to a low number of non-exclusively breastfed children in this study population.

Ferritin concentrations dropped dramatically between 3–4 months of age, as also observed by others. [6] Length and LAZ scores were predictors of low ferritin levels and were weakly and indirectly associated with hemoglobin concentrations. Therefore, rapid growth makes infants and young children a particular risk group for IDA when dietary iron supply is low. [3,4,33,38]

Ferritin levels showed a significant negative correlation with the consumption of ASF in the past 24 hours. This leads to the assumption that either the amount of the reported ASF was inadequate, or the children’s diet as such was high in phytates or polyphenols, which reduce the absorption of iron, or there are different causes of IDA in this age group such as parasites or hemoglobinopathies that had not been assessed in this study. [1,38–40] In fact, inherited hemoglobin disorders are among the main causes of anemia besides iron, vitamin A, or other micronutrient deficiencies, inflammation or chronic infections in Asian developing countries. [1,3,41]

When looking at optimal feeding practices, the results reveal that a CFI was not significantly and directly associated with hemoglobin but the effect of the interaction between sTfR and CFI on hemoglobin was significant. The more optimal feeding practices were applied, the less strongly sTfR and hemoglobin were correlated. This could be regarded as evidence that iron deficiency plays a minor role of iron deficiency in causing anemia among children who were fed according to the IYCF recommendations.

It was estimated that about 30% of anemia in young children in Cambodia is due to iron deficiency. [40] Low birth weight due to prematurity or maternal anemia is a known cause of iron deficiency prior to 6 months of age [40,42] Current literature also suggests that maternal hemoglobin status during pregnancy is a major determinant of child anemia and child birth weight. [43,44] Increasing evidence is provided on the role of timing of umbilical cord clamping on maternal and infant’s iron status for up to 6 months after birth. A Cochrane review highlighted positive effects of delayed clamping on hemoglobin concentrations and iron stores. This study is limited by the lack of data on this. [45,46] Nonetheless, iron deficiency below 6 months of age using two different cut-offs highlighted the need for further investigations on reliable cut-offs for this age group. [25]

As there is little information about the metabolism and interaction of biomarkers of iron and vitamin A status in infants below 6 months of age, the need for further blood analysis is highlighted. There is still uncertainty about the immunological responses, including acute phase response, which is not yet fully developed in young infants, and iron shift to and from the liver.

In the children examined, vitamin A supplementation rates were high and prevalence of vitamin A deficiency was found to be low, thereby, confirming results from another study in Cambodia. [41] Suboptimal vitamin A status was found to be a predictor of hemoglobin concentration as in previous studies [11,41,47,47]. In this Cambodian sample children who received a vitamin A supplement had a significantly higher RBP. In a moderator analysis with ferritin, vitamin A did not have a significant impact on the interaction between ferritin and RBP and their effect on hemoglobin. The impact of vitamin A supplementation in school children on their iron status was tested in Morocco and shown to significantly reduce anemia without changing total body iron, hence reducing available iron. [48] Another study from Thailand observed an impact of vitamin A supplementation to children aged 3–6 months on their hematological condition, thereby showing increased RBP and hemoglobin concentrations but no changes in ferritin. [49] However, vitamin A might have an effect on the absorption of iron and the risk of infection [47,48,50]. Neither were analyzed in this study due to appropriate inflammation adjustments not being available. In comparison to vitamin A supplementation the number of children who received a deworming tablet in the six months prior to the survey was low. The association between deworming status and hemoglobin levels was not tested in this sample. However, a current study from Lao PDR and Cambodia questioned the effect of a single dose of mebendazole on the prevention of parasitic infections. [51,52]

The analyses revealed low rates of inflammation as well as typical patterns of levels of ferritin and sTfR which were as expected and reported by others for this age group. [10,23,25] Sex of the child had no significant impact on any of the biomarkers as also determined in other studies. [3,25]

Besides a lack of data on hemoglobin disorders and parasitic infections, this study is limited by its cross-sectional design which does not permit any causal interference by time. The calculations were done using the continuous variables as the literature still lacks appropriate cut-offs, especially for infants. The cut-offs used were the most common ones, but they might not be suitable for this young age group. [25] However, results of anemia prevalence concur with the results of the latest CDHS from 2010. [13]

### Conclusion

In summary, ferritin and sTfR are strongly associated with hemoglobin status in this Cambodian sample. However, causes of anemia and IDA in Cambodian infants especially under 6 months of age still remain unclear, and further data collection is urgently needed in order to better design, direct, and implement nutrition programs in the country. To better understand the relationship between the iron status of infants under 2 years of age, it is of greatest necessity to also assess other micronutrient deficiencies, parasitic infections, lead toxicity, and the prevalence of hemoglobinopathies.

## Supporting Information

S1 Data Cambodia minimal data set. (XLSX)

S1 Fig Flow diagram. (DOC)

S1 Text Study protocol. (PDF)

S2 Text Trend statement checklist. (PDF)
