# Supplementary material for: Role of Breastfeeding and Complementary Food on Hemoglobin and Ferritin Levels in a Cambodian Cross-Sectional Sample of Children Aged 3 to 24 Months
Source: PLoS One. 2016 Mar 14;11(3):e0150750. doi: 10.1371/journal.pone.0150750 (PMC4790902; doi:10.1371/journal.pone.0150750)
Supplement: S1 Fig — (DOC) [file pone.0150750.s002.doc]

**
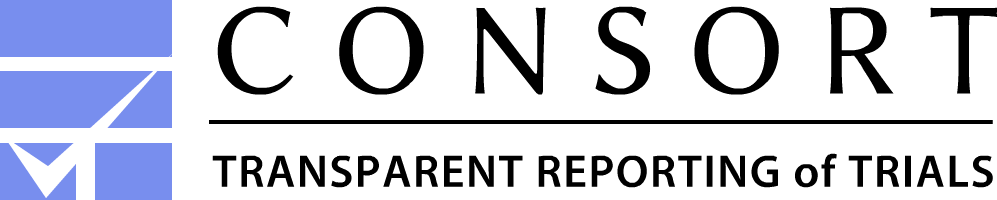
**

**CONSORT 2010 Flow Diagram**

**Analysis**

**Enrollment**

Randomly selected (n=1106)

Drop-Out (n= 74)

♦  Not meeting inclusion criteria (n= 38)

♦  Declined to participate (n= 2)

♦  Other reasons (n=34)

Participants (n= 1032)

Approx. number of children under 2 eligible (n=15000)

Final baseline sample (n= 1028)

Excluded (n= 4)

♦  Not meeting inclusion criteria (n= 1)

♦  second twin (n= 3)

Eligible for blood analysis (n= 928)

Excluded (n= 104)

♦  below 3 months of age (n=100)

♦  incomplete data (n= 4)
