# Supplementary material for: Role of Breastfeeding and Complementary Food on Hemoglobin and Ferritin Levels in a Cambodian Cross-Sectional Sample of Children Aged 3 to 24 Months
Source: PLoS One. 2016 Mar 14;11(3):e0150750. doi: 10.1371/journal.pone.0150750 (PMC4790902; doi:10.1371/journal.pone.0150750)
Supplement: S1 Text — (PDF) [file pone.0150750.s003.pdf]

# Effectiveness of a nutrition education intervention to improve complementary feeding practices in Cambodia: a restricted randomized trial

Justus Liebig University
Prof. Michael B. Krawinkel, MD
Dr. Irmgard Jordan
Dipl. oec. troph. Anika Reinbott
Dr Ouke Vanna, MD

**June 2012**

JUSTUS-LIEBIG-
UNIVERSITÄT
GIESSEN

FACHBEREICH 09
Agrarwissenschaften,
Ökotrophologie und
Umweltmanagement

## Table of contents

Abbreviations ........ I

1 Abstract ........ 1

2 Background and justification ........ 2

2.1 Trial of Improved Practices ........ 2

2.2 Background information Cambodia ........ 4

2.2.1 MALIS Project in Cambodia ........ 5

2.3 Rationale of the study ........ 7

3 Literature Review ........ 7

3.1 Malnutrition and the role of food security for improving nutrition ........ 7

3.2 The importance of complementary feeding ........ 9

4 Hypothesis ........ 10

5 Objectives ........ 11

6 Methodology ........ 11

6.1 Study sites ........ 11

6.2 Participants ........ 12

6.3 Study design ........ 13

6.3.1 Overview of the study design ........ 13

6.3.2 Trial design ........ 13

6.3.3 TIPs and Intervention ........ 14

6.4 Sample size for baseline and impact survey ........ 14

6.5 Randomization procedure ........ 15

6.6 Statistical methods and analysis ........ 16

6.7 Ethical considerations ........ 16

6.7.1 Recruitment, risks and benefits ........ 16

6.7.2 Informed consent ........ 17

6.8 Data collection ........ 18

6.8.1 Interview ..... 18
6.8.2 Anthropometric measurements ..... 18
6.8.3 Date of birth ..... 19
6.8.4 Blood samples ..... 20
6.8.5 Motor milestones ..... 20
6.8.6 Data collected Intervention ..... 21
6.9 Quality assurance ..... 21
6.9.1 Statement of Compliance ..... 21
6.9.2 Translation of questionnaire ..... 21
6.9.3 Recruitment of field staff ..... 21
6.9.4 Training and monitoring ..... 22
6.9.5 Assurance of communication ..... 22
6.9.6 Pretest ..... 23
6.9.7 Registration of the study ..... 23
6.10 Data protection ..... 23
7 Dissemination of findings ..... 24
8 Personnel ..... 25
9 References ..... 26
10 Annexes ..... 34

## Abbreviations

| | |
|---|---|
| AED | Academy of Educational Development |
| AGP | Acid glycoprotein |
| Aids | Acquired immune deficiency syndrome |
| ARI | Acute Respiratory Infection |
| CRP | C-reactive protein |
| EU | European Union |
| FAO | Food and Agriculture Organization of the United Nations |
| Fe | Iron |
| FFP | Food Facility Project |
| FSTP | Food Security Thematic Program |
| GIZ | German Society for International Cooperation |
| HAZ | Height for Age z-Score |
| HH | Households |
| HIV | Human Immunodeficiency Virus |
| IMCF | Improving the dietary intakes and nutritional status of infants and young children through improved food security and complementary feeding counseling |
| IRB | Institutional Review Board |
| MAFF | Ministry of Agriculture, Forestry and Fisheries |
| MALIS | Improving Food Security and Market Linkages for Smallholders project |
| MDG | Millennium Development Goals |
| MTCT | Mother-to-Child Transmission |
| NCHS | National Center for Health Statistics (US) |
| NGO | Non-governmental organizations |
| RBP | Retinol Binding Protein |
| RDA | Recommended Dietary Allowances |
| SMART | Standardized Monitoring and Assessment of Relief and Transitions |
| SUN | Scaling up nutrition |
| TAC | Technical Advisory Committee |
| TfR | Transferrin Receptor |
| TIPs | Trials of Improved Practices |
| UNICEF | United Nations International Children’s Emergency Fund |

| | |
|---|---|
| USAID | United States Agency for International Development |
| Vit A | Vitamin A |
| WAZ | Weight for Age z-Score |
| WHO | World Health Organisation |

# 1 Abstract

Undernutrition remains one of the biggest challenges in developing countries. Children aged 0-23 months are the most vulnerable group with a peak incidence of mortality and morbidity. The Cambodian Demographic and Health Survey 2010 estimated a national stunting prevalence of 40%. Results show that stunting increases after six months of age when most children receive complementary feeding.The promotion of a nutrient-dense diet based on locallyavailable foods is essential to improve the nutritional status of young children. In order to optimize infants and young children’s diets, the FAO project “Improving food security and Market Linkages for Smallholders in OtdarMeanchey and PreahVihear Provinces (MALIS)” will implement a food security project with a nutrition education component.

The objective of the research is toevaluate the effectiveness of the behavior change messages which will be delivered through a community based intervention involving community based organizations. The research design encompasses both qualitative and quantitative methods, including two cross-sectional surveys, focus group discussions and key informant interviews. The first cross-sectional survey will take place prior to the intervention to assess the baseline nutrition security situation of families with children below two years in the MALIS project region. The behavior change communication strategies will be implemented in the prior selected intervention areas by the MALIS project. The second cross-sectional surveywill be conducted after at least 18 months of intervention evaluating the impact of the intervention by a research team.

The primary outcome parameter to be measured will be mean Height for Age z-Scores (HAZ). Secondary outcome parameters will be vitamin A deficiency, irondeficiency and behavior change. Methods for data collection will includeanthropometric measurements, questionnaires, collection of blood plasma by finger prick and focus group discussions. Statistical analysis will compare primarily the difference ofthe mean height-for-age z-score of children in intervention and control areas.

# 2 Background and justification

## 2.1 Trial of Improved Practices

In order to support countries in their efforts to address problems of food insecurity and malnutritionFAO has been promoting improved complementary feeding in several countries[1] in the past years by teaching families how to enrich young children's diets using locally available nutrient-dense foods. InAfghanistan and Zambia FAO has assisted the Ministries of Agriculture and Health to improve complementary feeding through formative research using Trials of Improved Practices (TIPs – see box below). This approach has been especially set up by FAO to identify improved recipes and ideal messages for programming to improve feeding practices of women or caretakers of infants and young children. While all complementary feeding recipes and recommendations developed during TIPs are targeted to children 6 month of age and higher, it is important to note that exclusive breastfeeding up to 6 month of age is recommended during the TIPs process (1). It is part of FAO's efforts to integrate nutrition into agricultural and rural development activities and to foster linkages between the health and agricultural sector. Recommended dietary practices (including improved local recipes) have now been published in separate manuals for Afghanistan (2) and Zambia (3). Participatory cooking demonstrations using nutritionally improved recipes in conjunction with dietary counseling at household level to optimize young children's dietary intake have been introduced in various community development projects in these countries. FAO programs using the TIPs approach to improve infant and young child and family nutrition are ongoing in Malawi, Laos and Cambodia.

---

[1] Afghanistan, Cambodia, Lao People's Democratic Republic and Zambia

**Box: What are Trials of Improved Practices (TIPs)?**
TIPs are a formative research technique used in programs that promote change. The methodology has been well tested and validated, particularly with regard to health and hygiene behavior, and has been used in various countries to develop nutrition behavior change communication strategies, including infant and young child feeding practices(4-6).
Using TIPs,program planners gain an in depth understanding of families' preferences and capabilities, as well as the obstacles they face in improving their nutrition and their motivations in trying newbehaviors and practices. TIPs therefore allow program planners to pre-test, adapt and evaluate the actual practices and recommendations in line with local circumstances and needs for eventual dissemination and promotion on a larger scale.
The TIPs can be divided into the three steps:

1. Investigation of food security, family feeding and child feeding practices, development of preliminary list of improved feeding recommendations and training of TIPs facilitators,
2. TIPs implementation: participants explore how to improve their child's health and nutrition,
3. TIPs evaluation and development of detailed plan for disseminating acceptable and feasible feeding recommendations and recipes

Preliminary evidence from FAO programs that have employed TIPs to improve infant and young child feeding have demonstrated the following:

- families' interest in using an increased variety of locally available nutrient-dense foods accessible in different seasons to improve the nutritional adequacy of complementary foods,
- the relevance of introducing basic nutrition, child feeding and food selection and preparation skills, and
- the acceptability and practical feasibility of using improved complementary feeding recipes in the family setting.

The recipes were designed to meet the Recommended Daily Allowances (RDA) of children 6 to 23 months[2]with affordable locally available foods using the WHO Guiding Principles for Complementary Feeding ofthe Breastfeed Child (1). However, due to the absence of rigorous impact evaluation studies, up to nowthere is only

---

[2] Currently zinc recommendations for children 12-24 month cannot be met with locally available foods. Further clarification is needed why recommendations are up to 10 x higher for 12-24 moth old children compared to 6 11 month old children.

anecdotal evidence of the impact of these improved complementary foods and feedingrecommendations on feeding practices, children's dietary diversity, nutritional intake andanthropometric status identified through TIPs within a FAO project.

## 2.2 Background information Cambodia

According to the Global Hunger Index report 2011[3](7) Cambodia was able to improve its status from extremely alarming to serious. With 28% of the population living below the international poverty lineof 1.25 US$ per day, Cambodia remains vulnerable to natural disasters, volatile food prices and following this food insecurity (7, 8). The coverage with sanitation facilities and the access to improved drinking water sources continues to be low with 18% of the rural population using improved sanitation facilities and 61% using improved drinking water sources. The literacy rate among the 15 to 24 years old in the population is almost 90% (8).

Globally more than one third of child deaths are related to undernutrition (9, 76). In Cambodia the under-five mortality rate declined since 1990 but still remains high with 51 deaths per 1,000 live births in 2010 (8). In the Southeast Asian region, Cambodia shows higher prevalence of stunting, wasting and underweight among infants than other countries in the region (9). Rural Cambodian children under five show a higher prevalence for underweight with 30% than urban children with 19% (8). Factors contributing to child undernutrition are among others low birth weight[4], infectious diseases, maternal underweight and insufficient breastfeeding and complementary feeding practices. (10, 74-76)

Focusing on anthropometric data of length/height for age (Height-for-Age Z-Scores), the values decrease dramatically after two months of age (11, 12). Around the globe Asian countries show the most intense stunting rates with a mean value between 1.5 to 2SD below the WHO Growth Standard (13).According to the Cambodian Demographic and Health Survey 2010 (10) a total of 40% of children are stunted. 46% are younger than two years and 10% of the stunted children are younger than six months (Table 1).

---

[3] The Global Hunger Index (GHI) 2011 refers to data collected from 2004 to 2009. The GHI categorizes countries by giving scores for the proportion of undernourished in the population, child mortality and child underweight. The scores then allow a distribution of the countries according to their hunger situation into five groups: low, moderate, serious, alarming, and extremely alarming.

[4]Low birth weight is defined by the WHO as a weight below 2500g, for a given time period. (74)

**Table 1: Prevalence of stunting of children < 5 years in Cambodia**

| DHS 2010 | Prevalence of stunting (%) (Height for age Z-Score <-2SD)[1] | | n |
|---|---|---|---|
| | HAZ <-3SD | HAZ<-2SD[2] | |
| Children < 5 years | 13.6 | 39.9 | 3,975 |
| < 6months | 5.1 | 10.4 | 294 |
| 6-8 months | 5.9 | 20.5 | 230 |
| 9-11 months | 6.2 | 18.7 | 191 |
| 12-17 months | 9.3 | 32.0 | 412 |
| 18-23months | 13.7 | 46.7 | 414 |

[1] WHO Child Growth Standards 2006 (13)

[2] Includes children who are below -3 SD from the WHO Child Growth Standards population median

Following the WHO guidelines for complementary feeding of the breastfed and non-breastfed child (1, 73), a child should be exclusively breastfed for the first six months of life and from six months to 23 months a child should be given complementary foods in addition to breastmilk. This period in life is often referred to as a critical window because inadequate nutritional diets affect growth and vulnerability towards illness (5, 11, 76).Important factors are the early initiation of breastfeeding and the continuation ofexclusive breastfeeding up to six months (14). The complementary diet should consist of at least four out of the seven food groups each day (dietary diversity) andshould be at least fed for the minimum number of times per day (minimum meal frequency). Dietary diversity and minimum meal frequency result in a necessary minimum acceptable diet for children from six to 23 months. According to the CDHS 2010 73.5% of children below six months are exclusively breastfed. Almost 10% received breastmilk and plain water. The mean duration of exclusive breastfeeding in Cambodia is 4.9 months.96% of breastfed children from six months to two years of age were given solid or semisolid complementary foods in addition to breastmilk. Complementary food in Cambodia is mainly a watery rice-based porridge called *borbor*(14).

Less than one-third of the under twos in Cambodia meet the minimum criteria for dietary diversity and only about 50% received the minimum number of mealsaccording to the WHO guidelines(1,10).

### 2.2.1 MALIS Project in Cambodia

In 2012the ‘Improving Food Security and Market Linkages for Smallholders (MALIS)” projectwill be launched in two provinces in Northern Cambodia, OtdarMeanchey and

PreahVihear, as a further continuation of the FAO EU Food Facility Project from 2009 which was implemented in ten provinces in the country. Funded by the Food Security Thematic Program(FSTP) of the EU the “Improve the Food Security of Farming Families Affected by Volatile Food Prices” project was implemented from 2009 to 2011 in 50,000 farming households who were affected by volatile food prices. The project objective was based on five aims:

- Improved agricultural productivity
- Reduce post-harvest losses
- Improve fish production and fish resources management
- Improved water resources and irrigation
- Improve dietary diversity and family feeding (focus on Infant and young children)

The target group consists of vulnerable rural families who are depending primarily on agriculture for their livelihood.With the overall objective to improve the food security and nutrition situation of the target group, a close collaboration between the Ministry of Agriculture, Forestry and Fisheries (MAFF) and local NGOs is planned. The program addresses some of the countries objectives towards food security and agricultural development as well as the Millennium Development Goal 1 and 3 (MDG) to eradicate hunger and extreme poverty in Cambodia(16).The enhancement of income, agricultural productivity and nutritional status are among the main objectives as well as the promotion of the resilience to external shocks for vulnerable smallholder farmers in targeted provinces.

As one fourth of the Cambodian population is undernourished (17), the former project also includeda nutrition component. Following the TIPs formative research technique (see Chapter 2.1), the project paid special attention to the lack of knowledge in Infant and Young Child Feeding (IYCF) Practices. TIPs was conducted in 360 households of 4 villages in each of the nine provinces, one trial in the wet season (Aug - Sept 2010) and one trial in the dry season (Dec 2010 - Jan 2011). Each trial was followed by a cooking demonstration for at least 9,000 households. As a result of TIPs culturally acceptable complementary feeding practices were collected and are available for dissemination.

With a new fund from the FSTP of the EU the present MALIS projectis implemented in previous and new areas of two former target provinces,OtdarMeanchey and

PreahVihear. These provinces are considered to have a best fit to the framework established by the EU for targeting assistance under the certain program.

The expected outcome of MALIS nutrition component is that the capability of target households is to be strengthened to the extent that the diets of children below two years meet their nutritional needs by the end of the project.

## 2.3 Rationale of the study

FAO needs to document the impact of the TIPs approach, capitalize on lessons learnt and refine recommendations to inform the design of future interventions and prioritize resource allocation. However, FAO is not a research institution and has therefore asked the Justus Liebig University to evaluate their TIPs approach within ongoing FAO food security projects i.e.MALIS in Cambodia as well as the FAO/FICA project in Malawi. The expected outcome is an assessment of the effectiveness of promoting locally-available and affordable complementary foods to improve infant and young children's nutritional status based on TIPs within FAO food security projects.

# 3 Literature Review

## 3.1 Malnutrition and the role of food security for improving nutrition

The burden of undernutrition in many developing countries continues to be high and slows the potential for individual, social and economic development. High rates of wasting and stunting among children under-five years of age are a reflection of the serious challenges many developing countries are facing: inadequate access to and availability of healthy and diverse foods, improper feeding and caring practices, as well as poor health and hygiene conditions (18-21). The food and financial crises of 2008 and 2009 have brought governments' attention to the importance of addressing food and nutrition security as a fundamental component of socio-economic development and political stability. This trend is reflected by efforts to reform the Committee on World Food Security[5], the creation of the High-Level Task Force on

[5] The Committee for World Food Security (CFS) is the United Nations' forum for reviewing and following up on policies concerning world food security. It also examines issues which affect the world food situation. It was established as a result of the food crisis of the 1970s, upon recommendation from the 1974 World Food Conference.

Food Security[6] as well as donors' renewed interest in food and nutrition security (EU Food Facility; Spanish MDG-Fund on Children, Food Security and Nutrition; USAID's Feed the Future; World Bank Scaling Up Nutrition (SUN) Framework, and the 63rd World Health Assembly Resolution on Infant and Young Child Feeding). Growing attention is given to the role of agriculture and to the linkages between agriculture and health in improving nutrition.

The conceptual framework of nutrition (figure 1) shows the different impact factors on nutrition security as well as food and health security, which may be applied on individual, regional and national level.

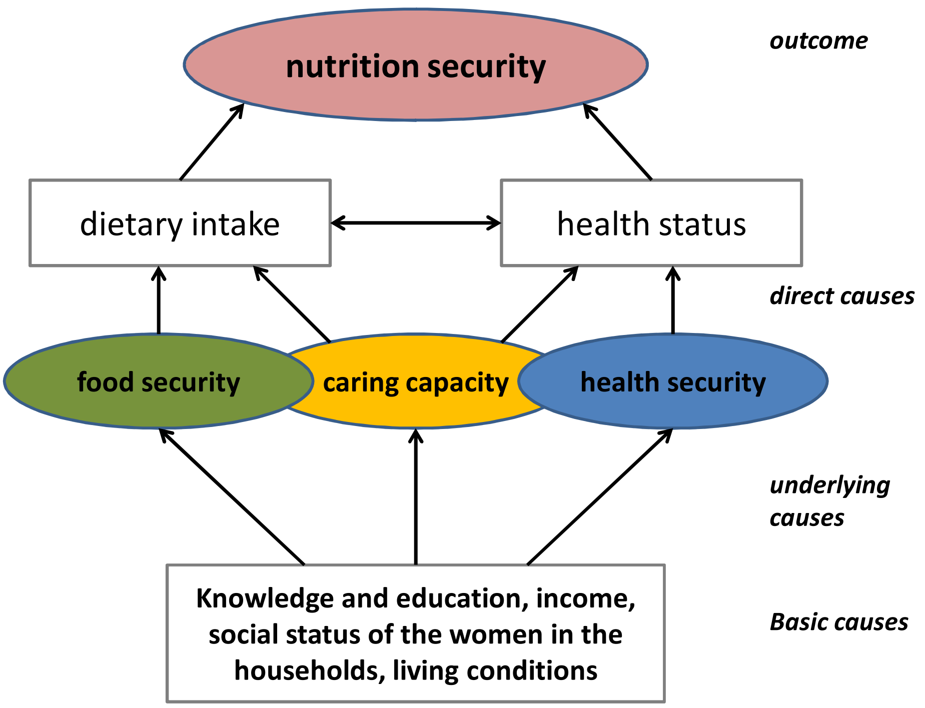


Figure 1: Adopted UNICEF framework of underlying causes of malnutrition and mortality (22,23).

The linkage between food production and nutritional health can only be achieved if the interactions among food diversity on the plate, people's nutritional knowledge and preferences, socio-economic factors, and crop diversity are considered (24).

---

[6] At the end of April 2008 the United Nations' Chief Executives Board established a UN System High Level Task Force (HLTF) as a temporary measure to enhance the efforts of the UN system and International Financial Institutions in response to the Global Food Security Crisis.

There has been a demand for in depth research in these areas for many years (25) in order to inform policy makers and programrs to ensure funds made available for nutrition interventions are optimally utilized (26).

## 3.2 The importance of complementary feeding

Several reviews of nutrition interventions (11, 12, 27, 28) have shown that increased attention needs to be given to complementary feeding interventions targeted to children aged 6-23 months, which is the period with the peak incidence of growth faltering, micronutrient deficiencies and infectious diseases in developing countries (11). The effects of poor nutrition resulting in stunting may also be associated with delayed motor and mental development (29, 30). Therefore, effective interventions that are preventing and reducing stunting during this vulnerable period should be a high priority.

Several interventions, targeting this age group, put the emphasis mainly on micronutrient supplementation and food supplements, as well as therapeutic feeding and care (31-46). A metaanalysis looking at the impact of micronutrient interventions revealed a limited positive effect on height gain per year with less than 0.1 cm/year through either single or multiple nutrient supplementation compared to controls (40). However, studies focusing on complementary feeding support and educational strategies have shown to have a larger impact. Pooled analysis of three studies focusing on nutritional education in food-secure populations showed an increase in mean Height for Age Z-score (HAZ) of 0.25 (95% CI 0.01-0.49) compared to the control group. The effect was even higher in studies in food insecure populations receiving food supplements with or without education (HAZ change 0.41, 95% CI 0.05-0.76) (12). An evaluation study in Haiti comparing the impact of recuperative and preventive health services to comparable households in the Haiti Demographic Health survey on linear growth confirmed these results. In addition, the study showed that the preventive program had had a greater impact than the recuperative program compared to the respective control group (mean HAZ difference: 0.341; 95% CI 0.104, 0.577 and mean HAZ difference: 0.183; 95% CI: -0.022, 0.388 respectively) (47).

Comprehensive food-based approaches that promote a variety of nutrient-dense local foods to improve children’s dietary intake and nutritional status are essential (18,48). Food-based approaches that focus on the use of locally available,

affordable, and accepted nutrient-dense foods and recipes are designed to empower local populations to optimally use their resources and limit their dependency on external resources. Therefore, these approaches have a higher potential to improve child nutrition in the longer term compared to programs that mainly rely on donor funding and the distribution of micronutrients or food supplements.

## 4 Hypothesis

Assuming that behavior change messages on breastfeeding and complementary feeding practices as well as hygiene aspects as generated by the TIPs are widely accepted and out into practice by the mothers with children ages 0-23 months the data assessment will be based on the following hypotheses:

1. The Height for age Z-Score (HAZ) of children below 2 years will increase if TIPs recommendations and recipes are locally available and accepted. At conclusion of the intervention, there will be a difference of 15% of the HAZ between interventions and controls:

H0: μ HAZ (intervention) = μ HAZ (control)

H1: μ HAZ (intervention) >μ HAZ (control)

2. Secondly, prevalence of anemia, vitamin A deficiency as well as morbidity rates will be reduced:

a) Anemia (A) among children between 6 and 23 months, defined as hemoglobin level of < 11g/dl, will decrease by 25 %:

H0: P(A| intervention) = P(A| control),

H1: P(A| intervention) < P(A| control)

b) Retinol binding protein (RBP) levels will decrease by 15% among children with a C-reactive protein (CRP) level of < 12mg/l:

H0: μ RBP (intervention) = μ RBP (control),

H1: μ RBP (intervention) <μ RBP (control)

c) Prevalence of children with presumed acute respiratory infections and fever (ARI) and diarrhea (D) in the last two weeks prior the survey will decrease by 25%:

H0: P(ARI| intervention) = P(ARI| control),

H1: P(ARI| intervention) < P(ARI| control)

H0: P(D| intervention) = P(D| control),

H1: P(D| intervention) < P(D| control)

d) There will be differences in knowledge of age-appropriate complementary feeding (KCF) of children between caretakers measured using a knowledge score in interventions and controls.

H0: μ KCF (intervention) = μ KCF (control),

H1: μ KCF (intervention) >μ KCF (control).

e) TIPs formative research generates nutritionally improved, culturally acceptable and affordable recipes which result in improved feeding practices and food intake which will result in a higher dietary diversity score for the children.

There will be differences in dietary diversity (DD) between interventions and controls:

H0: μ DD (intervention) = μ DD (control),

H1: μ DD (intervention) >μ DD (control).

## 5 Objectives

The purpose of this study is to observe the impact of promoting improved complementary feeding recipes and messages developed through TIPs on children's nutritional status and identify changes in complementary feeding practices in the two districts, OtdarMeanchey and PreahVihear, in Cambodia. The overall aim of the study is to evaluate the effectiveness of the wider dissemination of improved infant and young child feeding practices developed by TIPs to improve child nutritional status. The primary objective of the study is to show that children below two years have improved Height for Age Z-scores (HAZ) after at least 18 months of complementary feeding intervention, compared to children in matched control areas. Secondary objectives are to investigate whether children in the intervention area have improved nutritional status measured by vitamin A and iron status and improved health status measured by incidence of acute respiratory infection (ARI) and diarrhea compared to children in matched control areas.

## 6 Methodology

### 6.1 Study sites

The intervention and control areas will be selected following the targeted area of the MALISproject in two provinces, OtdarMeanchey and PreahVihear, in Cambodia. OtdarMeanchey is divided in five districts and PreahVihear is divided in seven districts. The baseline survey will only include the target regions of MALIS.

## 6.2 Participants

Knowing that recommendations on exclusive breastfeeding are included into the behavior change strategies all households with children 0-23 months of age are eligible to participate in the surveys. During the total data assessment period, families with children with a WAZ or WHZ-score <-2SD or sick children will be send for nutrition counseling and/ or treatment according to the countries guidelines.

Inclusion and exclusion criteria for participating in the surveys are:

- being resident in the sampled area,
- having at least one child 0 – 23 month of age,
- being randomly selected,
- accepting that anthropometric measurements and blood samples will be taken.

Families with a child who does not have a written record of the child’s date of birth or the date is not known by anyone in the family or who’s age cannot be estimated based on a seasonal calendar of local events around one month will be excluded from the study (see chapter 6.8.3).

## 6.3 Study design

### 6.3.1 Overview of the study design

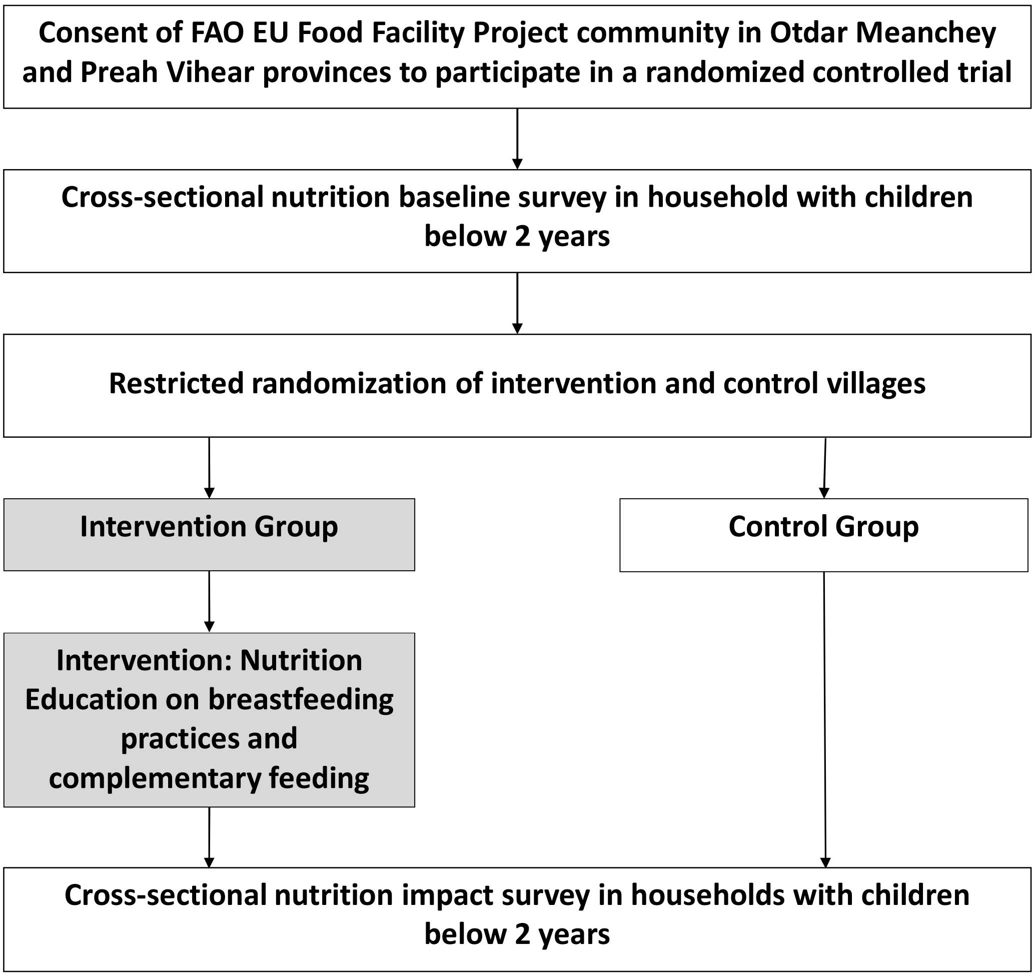


### 6.3.2 Trial design

A cross-sectional nutrition baseline survey targeting household with children under the age of two years will be conducted to assess the outcome indicators prior to the intervention in the selected areas in September 2012. Based on the results of the baseline restricted randomization will be conducted to identify intervention and control areas. MALIS will disseminate nutrition education and improved complementary feeding recipes gathered from TIPs in the FAO EU Food Facility project in Cambodia in selected households in the intervention area. After at least 18 months of intervention of nutrition education and complementary feeding counseling which will be conducted by MALIS a cross-sectional impact survey will be conducted to evaluate, whether improved complementary feeding practices and recipes

developed by TIPs have had an impact on the nutritional status of children below two years. The impact survey is scheduled for 2015.

### 6.3.3 TIPs and Intervention

TIPs has already been conducted under the FAO EU Food Facility Project in nine provinces in Cambodia from 2009 to 2011 (see Chapter 2.2.1). The intervention will be a wider dissemination of the improved recipes and behavior change messages developed through TIPs. During the dissemination phase data collection will include monitoring data on the dissemination and roll out of the behavior change messages through MALIS.

## 6.4 Sample size for baseline and impact survey

The appropriate sample size for the cross-sectional baseline survey will be determined largely by three factors (52):

a) estimated prevalence of the variable of interest,

b) desired level of confidence, and

c) acceptable level of error.

Using ENA for Smart sample size calculator (49) and considering a population size of 15000 children under two in the surveyed area, 50% of stunting (key indicator), desired precision of +/- 5% and a design effect of 3 the sample size calculation results in 1124 children. Avillage is the cluster unit.

The cross-sectional impact survey sample size will be based on the formula proposed by Haynes andMoulton for cluster randomized trials Haynes and Moulton (50):

$$n = (z_{\alpha/2} + z_{\beta})^2 \frac{(\sigma_0^2 + \sigma_1^2)}{(\mu_0 - \mu_1)^2}$$

The sample size calculation resulted in 335 children for each treatment arm, considering a power of80%, confidence level of 95%, $\sigma_0$= 1.5 and $\sigma_1$=1.2 and estimated 15% increase of HAZ ($\mu_0$=-1.96, $\mu_1$=-1.66)[7].

Nevertheless, to adjust for intra-class correlation (ICC), a design-effect should be included. The designeffect (DEFF) is defined as:

---

[7]Values have been estimated from the available literature.

$$DEFF = 1 + ICC\,(m-1)$$

Considering that m=19 children per cluster (village) will be measured, and an estimated ICC of 0.03 is applied (see annex 10.3), the DEFF= 1 + 0.03*18=1.54. Multiplying the DEFF-value with the above calculation this results in 516 children below two for each treatment arm. Adding an extra 10% to account for drop-outs or non-responders results in a sample size of 568 children below two years for each treatment arm.

## 6.5 Randomization procedure

A simple randomization proportional to population per district will be conducted to select 75 survey villagesfor the baseline. In each village 15 households with children under two will be randomly selected. In case there are less than 15 households with children under two in the selected village, the missing number of households will be randomly selected from the closest neighbouring village. The intervention and control villages are assigned following a restricted randomization based on the results of the baseline survey.

This implies that randomization will take place after adjusting for certain variables that might interfere with variables of interest to the study. Variables of adjustment are:

- district officer
- level of stunting
- total village population
- average land holding size
- wealth status
- access to health posts, nutrition rehabilitation unit, or nutrition counseling by a NGO

The selected clusters will be the objective of randomization. Randomization will bedone using the Software package “Experiment” and the operation “randomize”. The “Experiment”-package is a software extension to the statistical software *R*©. It serves to design and analyze different types of randomized trials, including cluster randomized trials, block, or matched pair designed trials (60).The baseline and the impact survey will randomly select the study participants in the selected clusters.

## 6.6 Statistical methods and analysis

Data assessment and data analysis will follow the adopted UNICEF framework of underlying causes of malnutrition and mortality (see Chapter 3.1). Nutritional status will be interpreted as a result of household food security, i.e. access to food and food availability, adequate social and care environment, i.e. direct caring behaviors, women's role, status and rights, social organizations and networks, and functioning public health system, i.e. health environment and access to health.

The cross sectional surveys will be used to gather information of the above mentioned parameters before and after the intervention. The data will be entered into the SPSS editor (IBM SPSS Statistics version 20) and checked for inconsistencies by two individuals independently. Flagged values will be checked based on the filled questionnaires and if applicable the study participants will be approached to verify the result.

Statistical analysis will be performed using the statistical packages of IBM SPSS statistics (version 20). At first the variables will be tested for normal distribution, followed by a descriptive analysis of prevalence of malnutrition, anemia, vit-A deficiency, respiratory infections and fever (ARI), diarrhea, morbidity rates, dietary diversity, and knowledge of age appropriate feeding. The latter involves a development of a score to describe the knowledge about age appropriate feeding. Further, regression analysis will be applied looking for causalities of malnutrition in the studied area.

Focus group discussions will be based on a general interview guide approach. The interview guide will include subjects of implementation, acceptance and obstacles of TIPs. They will be analyzed by applying the content analysis according to Mayring (51) and facilitated by the use of MAXQDA©-software (53).

## 6.7 Ethical considerations

Ethical approval will be applied for at the Institutional Review Board (IRB) of the Faculty of Medicine of the Justus Liebig University Giessen and local National Ethics Committee for Health Research of the Ministry of Health, Cambodia, prior to commencement of the study.

### 6.7.1 Recruitment, risks and benefits

List of possible eligible participants will be obtained from official population's list.

There is no risk to the participants involved in the study.

Benefits to Participants: This study has an indirect benefit to the participants. The study does provide anopportunity for the participants to gain information about their and their children’s current nutritionaland health status. Participants will receive a “Participants-Card” including their and their children’shealth data available directly in the field (anthropometric data, age and hemoglobin level). The card willprovide information whether the anthropometric measurements or the measured hemoglobin levelindicate poor nutritional status. In case of abnormal results participants will be sent for nutritioncounseling/ or treatment according to the guidelines of Cambodia for treatment of anemia andmalnutrition.

### 6.7.2 Informed consent

Prior to the surveys, general consent of the EU FAO project communities to participate in this trial will be obtained. Written consent will be obtained by the individual household to be interviewed at the day of data assessment.

The Investigators will be responsible for ensuring informed consent is obtained before any protocol specific procedures are carried out. The decision of a participant to participate in the research study is voluntary and will be based on a clear understanding of what is involved. Participants will receive adequate oral and written information about the nature and purpose of the study, participation/termination conditions, and risks and benefits – appropriate Participant Information and Informed Consent Forms are provided in the Annex. The oral explanation to the participants will be performed by designated people (enumerators), and must cover all the elements specified in the Participant Information Sheet and Informed Consent Form.

The participants will be given every opportunity to clarify any points they do not understand and, if necessary, ask for more information. The participant will be given sufficient time to consider the information provided.

The enumerator and the participant have to sign and date the Informed Consent Form to confirm that consent has been obtained. The participant will be provided with a copy of this document.

## 6.8 Data collection

The data collection in the cross sectional surveys will be based on an adaptation of the SMART methodology (49). The SMART methodology includes a questionnaire designed to assess mortality, nutritional status and food security in crisis situations. The final questionnaire will consist of standardized modules extracted from the questions designed by the SMART initiative, FAO, WHO, GIZ, and the Manoff Group (1, 6, 49, 54,55-59). Thus, mothers/caretakers with children below two years will be interviewed about their socio-economic situation, food security, mothers' and children's food intake, care, time availability, access to health, water and sanitation, motor milestones, and access to FAO food security activities by trained enumerators. Anthropometric measurements will be taken from mothers, their children below two years of age, and, if possible from fathers, by a nutritionist especially trained in anthropometric measurements. Capillary blood samples will be taken from the surveyed mothers (Hb only) and children below two years to assess the micronutrient status (retinol binding protein (RBP), transferring receptor (TfR), hemoglobin as well as the morbidity status (C-reactive protein (CRP), acyl glycoprotein (AGP) by medical trained research staff.

### 6.8.1 Interview

After written consent (see consent form in annex) the caretaker/mother of the child will be interviewed face-to-face by trained enumerators. Depending on the given infrastructure in the villages, participants are either invited in advance to come to a central meeting point, e.g. community center or primary school, to do the interview, or enumerators are coming to the homestead of the participants. If the interviews are conducted centrally in a public place, privacy will be assured by keeping an adequate distance between the interviewed participants that only the enumerators will hear the answers. In case village lists are not available, and systematic sampling is not possible, the EPI method will be used (49). Participants will then be invited on the day of selection to come to central meeting point at a certain time to avoid any inconvenience by waiting.

### 6.8.2 Anthropometric measurements

A central weighing and measuring station will be installed in every village. After the interview, mothers/caretakers (if possible the father as well) and their children will be

send to the weighing and measuring station. Weight and height of children and adults will be measured according to an anthropometric protocol based on the WHO report “Physical status: the use and interpretation of anthropometry” (1995) (60).

Weight of children will be determined with the child wearing no clothing. Adults’ weights will be taken while wearing light indoor clothing and no shoes. Heights and weights will be assessed to the nearest 0.5 cm and 0.1 kg, respectively. To determine the nutritional status of pregnant women, mid-upper arm circumference (MUAC) will be measured. All measures will be taken twice and the mean value is used for analysis (see ISAK manual (61)).

Anthropometric measurements will be taken with standardized equipment from Seca (SecaGmbh& Co KG, Hamburg, Germany). Weight will be measured using standardized digital flatscales (Seca 874, capacity: 200 kg) with mother/child function. Infants and small children are weighed while being held by the mother. The weight of the mother is assessed separately. Then the Mother-Child function ascertains the tare of the weight. Recumbent length will be taken from children with measuring boards (Seca 417, measurement range: 10–100 cm).

The height of adults will be measured with a stadiometer (Seca 213, measuring range: 20 – 205 cm). A non-stretchable measuring tape will be used to take MUAC (Unicef). After the weight and length assessment the children will be tested for edema. Edema will be diagnosed by applying moderate finger pressure on the tops of the child’s feet. If there is edema, an impression will clearly remain for at least a few seconds (on both feet). Edema will be recorded as absent, mild (both feet/ankles), moderate (both feet/ankles plus lower legs, hands or lower arms) and severe (generalized edema including both feet, legs, hands, arms, and face) (49).

Two pairs of trained research staff, each consisting of a measurer and an assistant, will take all measurements. Privacy of the participants while taking their measurements will be assured.

### 6.8.3 Date of birth

Children’s dates of birth (age) will be recorded either from immunization cards or birth registration cards. If no documents are available and the mother/caretaker doesn’t know the child’s birth-date, the age willbe estimated based on the FAO Guidelines for Estimating the Month and Year of Birth of Young Children (FAO 2008) (62).

### 6.8.4 Blood samples

Blood samples will be taken at the central weighing and measuring station. A medical technical assistant from Cambodia will obtain the blood sample for the biochemicalparameters via a finger prick using sterile disposable micro lancets to obtain capillary blood.

The analysis of biochemical measurement will include assessment of retinol binding protein (RBP), hemoglobin, transferrin receptor (TfR), acyl glycoprotein (AGP) and C-reactive protein (CRP). RBP, TfR, AGP and CRP will be analyzed using sandwich ELISA technology (63). With this technology it is possible to combine the measurements of the four proteins (TfR, RBP, CRP, AGP). Instead of using four different ELISA methods with different chemicals and procedures only one method is used. This also reduces the necessary amount of blood. A plasma volume of 2 times 7.5 µl is sufficient to do a double measurement of all four proteins. Directly after taking the blood samples they will be centrifuged and serum/plasma is stored in 0.2 mL PCR tubes. These tubes can also be directly used in an automatic pipettor to avoid the tedious and error prone manual pipetting. Samples will be stored on ice in a high efficient styrofoam box with more than 5 cm thick walls and tightly closing lid. All blood samples will be stored on ice until the end of the survey. Analysis of blood samples will be done by Dr. JuergenErhardt, DBS-Tech, Germany.

Hemoglobin concentration will be assessed immediately at the field site with a portable HemoCueHb301* analyzer (HemoCue, Grossostheim-Germany) following operating guidelines (HemocueHb 301+ operating manual. HemoCue GmbH, Grossostheim, Germany).

### 6.8.5 Motor milestones

Motor milestones will be assessed according to the Motor Development Study component of the WHO Multicenter Growth Reference Study (MGRS) (63). The following six distinct gross motor milestones will be recorded: sitting without support, hands-and knees crawling, standing with assistance, walking with assistance, standing alone, and walking alone. To assess all milestones, the standardized testing procedures from MGRS will be adopted. A milestone will only be considered as achieved, if all given criteria are met.

#### 6.8.6 Data collected Intervention

During the intervention focus group discussions as well as open one-on-one interviews will be held with family members (especially women and elderly), health workers and nutritionists. Focus group discussions and one-on-one interviews are qualitative empirical research methods and imply that questions are posed in a colloquial manner instead of a standardized questionnaire. The focus groupdiscussions will follow a general interview guide approach. During the focus group discussion participants are encouraged to talk freely about the subject. This will enlarge the understanding of how behavior change messages have been integrated in daily life, how behavior has changed and will as well identify obstacles to the implementation. Therefore, focus group discussions will allow gaining deeper understanding of the complex research background. (65-67).

### 6.9 Quality assurance

#### 6.9.1 Statement of Compliance

The study will be conducted in accordance with the design and specific provisions of this InstitutionalReview Board (IRB) approved protocol. Good Epidemiological Practice (GEP) as recommended in the"Guidelines for proper conduct of epidemiological research" provided by the International Epidemiological Association (IEA).
The principal investigators will assure that no deviation from or changes to the protocol will take place without prior agreement from the sponsor and documented approval from the IRB. The principal investigator will promptly report to the IRB and the sponsor any changes in research activity and all unanticipated problems.

#### 6.9.2 Translation of questionnaire

Questionnaires will be designed in English and translated into Khmer by native speaking nutritionists. The translated questionnaires will be tested on three to five native speakers. To assure analogous translations, independent, native speaking nutritionists will translate both versions of the questionnaire back into English.

#### 6.9.3 Recruitment of field staff

Main requirements for enumerators are:

- language skills (Khmer)

- minimum of knowledge in nutrition related research and basic interview experiences

A total of 12 enumerators will be enrolled in the data collection process. Two medical trained research staff will be recruited from local hospitals to take blood samples and to assist the HemoCue analysis.

### 6.9.4 Training and monitoring

Enumerators: The enumerators will be trained on the questionnaire. A guideline on how to conduct the interview will be developed and used during the training workshop. Interviews will be conducted pairwise. One enumerator will ask the questions, the other one will record the answers. The training will as well include the correct assessment of motor milestones based on the observation criteria used in the WHO MGRS (64).

During the data collection process enumerator teams will be matched randomly every day. Interviewers will be systematically and frequently monitored and completed questionnaires will be controlled for missing data and consistency on a daily basis.

Anthropometric measurements: In order to assure that all measurements are taken in the same way the training for taking anthropometric measurements will be based on the WHO Child Growth Standards "Training Course on Child Growth Assessment" (68). Staff members of the JLU Giessen and Mahidol University will carry out the training. Furthermore the training curricula will cover issues of sensitivity to local customs, dress, and practices of modesty. Correct handling of equipment will as well be included in the training. To avoid problems in this area, observers of the same sex as the subjects will be employed. Since the research project mainly focuses on young children and their mothers/caretakers, women will be recruited to take the measurements. Survey leaders will frequently visit the weighing and measuring station. These visits will serve to verify that the anthropometry protocol is being implemented properly and consistently. Retraining sessions will be arranged when a lack of standardization is observed among the researchers.

### 6.9.5 Assurance of communication

Supervisors are responsible for the technical quality of the surveys for which the survey teams under their charge are conducting. Enumerators will be randomly assigned to a supervisor. One supervisor will be in charge of three survey teams.

The duties of a supervisor include advice and control of the accuracy of the survey data. The research team will include a translator for the research supervisors from Germany. This will assure the communication between all research team members as well as participants. Survey leaders and survey supervisors will record all important points in a notebook as soon as possible, including observations, ideas, problems, actions taken to address these problems, and the reasoning behind any decisions taken. Survey leaders, supervisors, and surveyors will meet daily after the survey implementation.

#### 6.9.6 Pretest

A pretest of the entire data collection process will be conducted in villages not selected for the baseline survey. The pretest serves to ensure that the questionnaire is fully understood by the enumerators as well as to test the enumerators' behavior in conducting the interviews. The results will be evaluated by the trainers and a final consultation is held with the enumerators. Difficulties encountered by the enumerators with the questionnaire will be discussed and eventually phrasing or translations will be adjusted. The aim is to clarify any remaining uncertain points and to reach an agreement on the final questionnaire to be used.

#### 6.9.7 Registration of the study

The IMCF research study will be registered at the German Clinical Trials Register (DRKS). The DRKS is an open access online register for clinical trials, which allows all users to search, register and share information on clinical trials. The DRKS is free and publicly accessible. The DRKS is an approved Primary Register in the WHO network since October 2008 and thus meets the requirements of the International Committee of Medical Journal Editors (ICMJE).

### 6.10 Data protection

Data management procedures will protect confidentiality of all data collected on individuals. All Investigators and study site staff involved with this study must comply with the requirements of the respective data protection laws in Cambodia and Germany with regard to the collection, storage, processing and disclosure of personal information. Access to collated participant data will be restricted to the survey management and stored in a locked cupboard.

Each subject will be assigned a unique identification code that will be used for data entry and analysis.To safeguard confidentiality, subject records are accessible only to the team doing the initial data entry, and the individual checking as a part of the dual entry system. Computers used to collate the data will have limited access measures via user names and passwords. Identity information and consent forms are not kept in the main computerized data file, but in a hard copy kept in a locked cabinet available only to the principle investigators. The front page of the individual record with consent and individual identity information is separated from the rest of the record, which contains only the individual code number.

Test tubes and specimens used by laboratory staff will be labeled by individual code numbers only. The collected blood samples will only be used for the specific purpose covered by the informed consent given.

Published results will not contain any personal data that could allow identification of individual participants.

## 7 Dissemination of findings

Reports on the project's progress and regular monitoring of the project activities will be provided by the FAO consultant, the Giessen PostDoc and the Principal Investigators. The research project team will be responsible to produce six-monthly progress reports, which will

- contain information on main activities and compliance with the work plan;
- identify any problems and constrains encountered during the research progress,
- provide recommendations for corrective measures;
- if necessary, revise the work plan for the following reporting period.

A contact information database will be created and maintained that will be used for group/individual mailings of paper documents and to facilitate telephone and fax communications between the Project Management, Site Management, and TAC. The project visibility will be enhanced by the launching of a FAO project website. The website will be updated at least on a 6 monthly basis.

- Lessons learnt from the project and research results will be shared through:participation and presentations in relevant conferences and technical consultations regarding nutrition and feeding of infants and young children,

- preparation of research articles to be submitted to scientific journals,
- documentation of case studies, and
- preparation of guidelines and technical recommendations on improved complementary feeding using local resources.

## 8 Personnel

The research has been developed and will be carried out by the Institute of Nutritional Sciences, Justus Liebig University Giessen, Germany in collaboration with Institute of Nutrition, Mahidol University, Bangkok, Thailand. A PhD student from Mahidol University and a PhD student from JLU-Giessen (AnikaReinbott) will undertake data collection and analysis; they will be supported by MSc students from Germany and Thailand and Cambodia.

Representatives from FAO (Ellen Muehlhoff), Mahidol University, Institute of Nutrition and JLU-Giessen (Dr. Irmgard Jordan) will be involved in project management and technical implementation. They will provide oversight of the project's implementation, approve workplans and associate budgets, and decide upon adjustments to the project implementation strategy as required. Furthermore, they will be responsible for ensuring that information is effectively shared between FAO, Giessen and national research institutes and governments, and between country-level teams and FAO headquarters and JLU Giessen in Germany.

A Technical Advisory Committee (TAC) has been formed including researchers and practitioners from academic institutions, UN partner agencies and NGOs having been involved in similar research and/or field work on complementary feeding, behavior change and nutritional impact studies. The TAC will provide advice on the research methodology, preliminary research results and other technical issues as they arise.

At country level, the official collaboration between IMCF research group (JLU and Mahidol University) and FAO EU project will start with a common meeting on national and district level organized by FAO in May (between 28th and 31st) in Siem Reap, Cambodia. Relevant government departments and district administration/representatives will be invited. A second meeting round will be held as soon as the results of the nutrition survey are available and the cluster randomization needs to be conducted.

# 9 References


1. PAHO. WHO | Guiding principles for complementary feeding of the breastfed child[Internet]. 2003 [cited 2011 Apr 13]; Available from:http://www.who.int/child_adolescent_health/documents/a85622/en/index.html
2. Dirorimwe C. Healthy Food, Happy Baby, Lively Family - Improved Feeding Practices and Recipes for Afghan Children and Mothers [Internet]. 2008; Available from: ftp://ftp.fao.org/docrep/fao/011/ak183e/ak183e.pdf
3. Dirorimwe C. Improved Complementary Foods Recipe Booklet Family Foods for Breastfed Children in Zambia [Internet]. 2007 [cited 2011 Apr 13]; Available from:ftp://ftp.fao.org/docrep/fao/010/ai208e/ai208e.pdf
4. Dickin K, Griffiths M, Piwoz E. Designing by dialogue. A Program Planner's Guide To Consultative Research for Improving Young Child Feeding. Washington, DC: Academy for Educational Development. 1997;
5. Muehlhoff, E., Dirorimwe, C., Huang, S., Mengkheang, K., &Ry, L. K. (July 2011). Trials of Improved Practices (TIPs) - Guiding notes for TIPs Trainers and Implementers. Available from: http://www.fao.org/docrep/014/am868e/am868e00.pdf
6. Muehlhoff, E., Dirorimwe, C., Huang, S., Mengkheang, K., &Ry, L. K. (2011, Juli). Trials of Improved Practices - Reference Notes and Tools - A manual for TIPs Trainers and Implementers. Available from: http://www.fao.org/docrep/014/am869e/am869e.pdf
7. IFRPI: von Grebmer, K., Torero, M., Olofinbiyi, T., Fritschel, H., Wiesmann, D., Yohannes, Y., Concern Worldwide and DWHH: Schofield, L., u. a. (2011, October). Global Hunger Index 2011 - The challenge of hunger: Taming price spikes and excessive food price volatility. Available from:http://www.welthungerhilfe.de/fileadmin/media/pdf/WHI/WHI2011/GHI-engl-2011_final.pdf
8. UNICEF. (2010). Cambodia - Statistics. [cited April 23, 2012]; Available from:http://www.unicef.org/infobycountry/cambodia_statistics.html
9. Anderson, V. P., Jack, S., Monchy, D., Hem, N., Hok, P., Bailey, K. B., &Gibson, R. S. (2008). Co-existing micronutrient deficiencies among stunted Cambodian infants and toddlers. Asia Pacific Journal of Clinical Nutrition, 17(1), 72 – 79.

10. National Institute of Statistics, M. of P. C., Directorate General for Health, M. of H. C., & MEASURE DHS, I. M. U. (2011, September). Demographic and Health Survey 2010. Available from: http://www.measuredhs.com/pubs/pdf/FR249/FR249.pdf
11. Victoria CG, de Onis M, Hallal PC, Blossner M, Shrimpton R. Worldwide Timing of Growth Faltering: Revisiting Implications for Interventions. PEDIATRICS. 2010;125(3):e473-e480.
12. Bhutta ZA, Ahmed T, Black RE, Cousens S, Dewey K, Giugliani E, et al. What works? Interventions for maternal and child undernutrition and survival. The Lancet. 2008; 371(9610):417-440.
13. WHO. WHO | WHO Child Growth Standards: Methods and development: Length/height-for-age, weight-for-age, weight-for-length, weight-for-height and body mass index-for-age. [Internet]. [cited 2011 Apr 18]; Available from: http://www.who.int/childgrowth/publications/technical_report_pub/en/index.html
14. FAO. (2011). Promoting Improved Complementary Feeding (with recipes). Abgerufen von http://www.fao.org/docrep/014/am867e/am867e00.htm
15. Lutter, C., Daelmans, B., de Onis, M., Kothari, M., Ruel, M., Arimond, M., Deitchler, M., u. a. (2011). Undernutrition, Poor Feeding Practices, and Low Coverage of Key Nutrition Interventions. Official Journal of the American Academy of Pediatrics, 128(6), 1418 – 1427.
16. UNDP (2008). MDG Monitor: Quick Facts Cambodia. MDG Monitor: Track, Learn, Support. [cited April 23, 2012]; Available from:http://www.mdgmonitor.org/country_progress.cfm?c=KHM&cd=116
17. FAOSTAT - World Hunger Map. (2012). [cited April 16, 2012]; Available from http://faostat.fao.org/site/563/default.aspx
18. Daelmans B, Mangasaryan N, Martines J, Saadeh R, Casanovas C, Arabi M, et al. Strengthening action to improve feeding of infants and young children 6 to 23 months of age: summary of a recent World Health Organization/UNICEF technical meeting, Geneva, 6-9 October 2008. In: WHO/UNICEF technical meeting on“Strengthening action to improve feeding of infants and young children 6-23 months of age in nutrition and child health programs”, Geneva, Switzerland, 6-9 October 2008. 2009. p. S236–S238.

19. Motarjemi Y, K\aferstein F, Moy G, Quevedo F. Contaminated weaning food: a major risk factor for diarrhoea and associated malnutrition. Bulletin of the World Health Organization. 1993; 71(1):79.
20. Brown K, Dewey K, Allen L. Complementary feeding of young children in Developing countries: a review of current scientific knowledge. Geneva: World Health Organization; 1998.
21. Boyce DG, Lewis MR, Worm B. 6th report on the world nutrition situation: PROGRESS IN NUTRITION. Nature. 2010; 466(7306):591–596.
22. Jonsson U. Strategy for Improved Nutrition of Children and Women in DevelopingCountries. A UNICEF Policy Review 1990-1. 1981;
23. Krawinkel MB. The value of Asian-Africa collaboration in food and health security. Asia Pac J ClinNutr. 2009; 18(4):570–576.
24. Keding GB. Linking nutrition security and agrobiodiversity: the importance of Traditional vegetables for nutritional health of women in rural Tanzania. 2010;
25. Engesveen K, Shrimpton R. Nutrition education in the context of the UnitedNations Standing Committee on Nutrition activities and publications, 1985-2006. J NutrEducBehav. 2007 Dec; 39(6):351-356.
26. Ruel MT. Can food-based strategies help reduce vitamin A and iron deficiencies? a review of recent evidence. International Food Policy Research Institute (IFPRI). 2001; (5).
27. Ashworth A, Ferguson E. Dietary counseling in the management of moderatemalnourishment in children. Food Nutr Bull. 2009 Sep; 30(3 Suppl):S405-433.
28. Shi L, Zhang J. Recent Evidence of the Effectiveness of Educational Interventions for Improving Complementary Feeding Practices in Developing Countries [Internet]. 2010 [cited 2011 Apr 12]; Available from: http://tropej.oxfordjournals.org/content/early/2010/06/16/tropej.fmq053.full.pdf+html
29. Gibson RS, Abebe Y, Hambidge KM, Arbide I, Teshome A, Stoecker BJ.Inadequate feeding practices and impaired growth among children from subsistencefarming households in Sidama, Southern Ethiopia. Matern Child Nutr. 2009 Jul;5(3):260-275.
30. Wu L, Katz J, Mullany LC, Haytmanek E, Khatry SK, Darmstadt GL, et al. Association between Nutritional Status and Positive Childhood Disability

Screening Using the Ten Questions Plus Tool in Sarlahi, Nepal. Journal of Health, Population, and Nutrition. 2010; 28 (6):585.

31. Krebs NF, Hambidge KM, Mazariegos M, Westcott JL, Goco N, Wright LL, et al.Complementary feeding: a Global Network cluster randomized controlled trial. BMCPediatr. 2011;11(1):4.
32. Kwena AM, Terlouw DJ, De Vlas SJ, Phillips-Howard PA, Hawley WA, FriedmannJF, et al. Prevalence and severity of malnutrition in pre-school children in a rural area of western Kenya - Kwena et al. 68 (4 Supplement): 94 -- American Journal of Tropical Medicine and Hygiene [Internet]. 2003 [cited 2011 Apr 12]; Available from:http://www.ajtmh.org/cgi/reprint/68/4_suppl/94
33. Phuka JC, Maleta K, Thakwalakwa C, Cheung YB, Briend A, Manary MJ, et al.Complementary Feeding With Fortified Spread and Incidence of Severe Stunting in6- to 18- Month-Old Rural Malawians. Arch PediatrAdolesc Med. 2008 Jul 1;162(7):619-626.
34. Lin CA, Manary MJ, Maleta K, Briend A, Ashorn P. An Energy-Dense Complementary Food Is Associated with a Modest Increase in Weight Gain When Compared with a Fortified Porridge in Malawian Children Aged 6 18 Months. The Journal of Nutrition. 2008 Mar 1; 138(3):593 -598.
35. Owino VO, Amadi B, Sinkala M, Filteau S, Tomkins A. Complementary feeding practices and nutrient intake from habitual complementary foods of infants and children aged 6–18 months old in Lusaka, Zambia. African Journal of Food, Agriculture, Nutrition and Development. 2008; 8(1).
36. Adu-Afarwuah S, Lartey A, Brown KH, Zlotkin S, Briend A, Dewey KG. Home fortification of complementary foods with micronutrient supplements is well accepted and has positive effects on infant iron status in Ghana. The American Journal of Clinical Nutrition. 2008 Apr 1; 87(4):929 -938.
37. Ouedraogo HZ, Nikiema L, Some I, Sakande J, Dramaix-Wilmet M, Donnen P. Home-based practices of complementary foods improvement are associated with better height-for-age Z-score in 12-23 months-old children from a rural district ofBurkina Faso. African Journal of Food, Agriculture, Nutrition and Development. 2008;8(2):204–218.
38. Guyon AB, Quinn VJ, Hainsworth M, Ravonimanantsoa P, Ravelojoana V, Rambeloson Z, et al. Implementing an integrated nutrition package at large scale

in Madagascar: The Essential Nutrition Actions Framework. Food &# 38; Nutrition Bulletin. 2009;30(3):233–244.

39. Phuka JC, Maleta K, Thakwalakwa C, Cheung YB, Briend A, Manary MJ, et al.Postintervention growth of Malawian children who received 12-mo dietarycomplementation with a lipid-based nutrient supplement or maize-soy flour. The American Journal of Clinical Nutrition. 2009 Jan 1;89(1):382 -390.
40. Ramakrishnan U, Nguyen P, Martorell R. Effects of micronutrients on growth of Children under 5 y of age: meta-analyses of single and multiple nutrient interventions. American Journal of Clinical Nutrition. 2008;89(1):191-203.
41. Defourny I, Minetti A, Harczi G, Doyon S, Shepherd S, Tectonidis M, et al. A Large-Scale Distribution of Milk-Based Fortified Spreads: Evidence for a New Approach in Regions with High Burden of Acute Malnutrition. PLoS ONE. 2009;4(5):e5455.
42. Macharia-Mutie CW, Brouwer ID, Mwangi AM, Kok FJ. Complementary feeding Practices and dietary intake among children 12-23 months in Mwingi district, Kenya. 2010 [cited 2011 Apr 12]; Available from:http://inderscience.metapress.com/app/home/contribution.asp?referrer=parent&backo=issue,5,7;journal,1,5;linkingpublicationresults,1:121116,1
43. Chhagan MK, Van den Broeck J, Luabeya KKA, Mpontshane N, Tomkins A, Bennish ML. Effect on longitudinal growth and anemia of zinc or multiple micronutrients added to vitamin A: a randomized controlled trial in children aged 6-24 months. BMC Public Health. 2010; 10(1):145.
44. Berti PR, Mildon A, Siekmans K, Main B, MacDonald C. An adequacy evaluation of a 10-year, four-country nutrition and health program. International Journal of Epidemiology. 2010 Apr 1; 39(2):613 -629.
45. Flax VL, Ashorn U, Phuka J, Maleta K, Manary MJ, Ashorn P. Feeding patterns ofunderweight children in rural Malawi given supplementary fortified spread at home. Maternal & Child Nutrition. 2007; 4(1):65-73.
46. Flax VL, Thakwalakwa C, Phuka J, Ashorn U, Cheung YB, Maleta K, et al. Malawian mothers' attitudes towards the use of two supplementary foods for moderately malnourished children. Appetite. 2009; 53(2):195–202.
47. Donegan S, Maluccio JA, Myers CK, Menon P, Ruel MT, Habicht JP. Two Food-Assisted Maternal and Child Health Nutrition Programs Helped Mitigate the

Impact of Economic Hardship on Child Stunting in Haiti. The Journal of nutrition. 2010;140 (6):1139.

48. Paul KH, Muti M, Chasekwa B, Mbuya MNN, Madzima RC, Humphrey JH, et al. Complementary feeding messages that target cultural barriers enhance both the use of lipid-based nutrient supplements and underlying feeding practices to improve infant diets in rural Zimbabwe. Maternal & Child Nutrition. 2010:no-no.
49. 48 Golden M, Brennen M, Brennan R. Measuring Mortality, Nutritional Status, and Food Security in Crisis Situations: SMART METHODOLOGY [Internet]. 2006;Available from: http://www.smartmethodology.org/images/stories/SMART_Methodology_08-07-2006.pdf
50. Hayes R, Moulton LH. Cluster randomised trials. Boca Raton: CRC Press; 2009.
51. RSeek.org R-project Search Engine [Internet]. 2011 Apr 18 [cited Apr18, 2011]; Available from: http://www.rseek.org/?cx=010923144343702598753%3Aboaz1reyxd4&newwindow=1&q=experiment&sa=Search+functions%2C+lists%2C+and+mo e&cof=FORID%3A11&siteurl=rseek.org%2F#1022
52. Mayring P. Qualitative Inhaltsanalyse Grundlagen und Techniken. 11th ed. Weinheim: Beltz; 2010.
53. MAXQDA - The Art of Text Analysis [Internet]. [cited 2011 May 5]; Available from:http://www.maxqda.de/
54. Manoff Group. Trials of Improved Practices (TIPs) Giving Participants a Voice in Program DesignTrials [Internet]. 2005 [cited 2011 Apr 18]; Available from: http://www.manoffgroup.com/resources/summarytips.pdf
55. WHO | Assessing the iron status of populations-second edition [Internet]. 2004 [cited 2011 Apr 14]; Available from: http://www.who.int/nutrition/publications/micronutrients/anaemia_iron_deficiency/9789241596107/en/index.html
56. WHO-Indicators for assessing infant and young child feeding practices: Part II Measurement [Internet]. 2010; Available from:http://www.who.int/nutrition/publications/infantfeeding/9789241599290/en/index.html

57. Gross R, Kielmann A, Korte R, Schoeneberger H, Schultink W. Nutrition baseline surveys in communities. Nutrition surveys and calculations: guidelines, software and additional information (Erhardt J, Gross R, eds.). Version current. 1997;
58. Deitchler M, Bellard T, Swindale A, Coates J. Validation of a measure of household hunger for cross-cultural use. 2010; Available from: http://pdf.usaid.gov/pdf_docs/PNADU857.pdf
59. FAO, Fanta. Guidelines for measuring household and individual dietary diversity [Internet]. 2008 [cited 2011 May 4]; Available from: http://www.foodsec.org/fileadmin/ user_upload/eufaofsi4dm/docs/guidelines_MeasuringHousehold.pdf
60. WHO Expert Committee on Physical Status: the Use and Interpretation of Anthropometry. Physical status : the use and interpretation of anthropometry : report of a WHO Expert Committee. Geneva: World Health Organization; 1995.
61. Marfell-Jones M, Olds T, Stewart A, Carter L. International standards for anthropometric assessment. Potchefstroom, South Africa: ISAK; 2006.
62. FAO, IFAD. Guidelines for Estimating the Month and Year of Birth of Young Children [Internet]. 2008 [cited 2011 Apr 14]. Available from:ftp://ftp.fao.org/docrep/fao/011/aj984e/aj984e00.pdf
63. Erhardt JG, Estes JE, Pfeiffer CM, Biesalski HK, Craft NE. Nutritional Methodology. 2004;
64. Wijnhoven TMA, de Onis M, Onyango AW, Wang T, Bjoerneboe G-EA. Assessment of gross motor development in the WHO Multicentre Growth Reference Study.pdf. 2004 [cited 2011 Apr 18]; Available from:http://www.who.int/childgrowth/mgrs/en/fnb_motor_37_45.pdf
65. Bortz J, Doering N. Forschungsmethoden und Evaluation für Human- undSozialwissenschaftler mit 87 Tabellen. 4th ed. Heidelberg: Springer-Medizin-Verl.; 2009.
66. Greenbaum T. Moderating focus groups: a practical guide for group facilitation. Thousand Oaks Calif.: Sage Publications; 2000.
67. Krueger R. Focus groups : a practical guide for applied research. 3rd ed. Thousand Oaks Calif.: Sage Publications; 2000.
68. WHO. Training course on child growth assessment. 2008 Geneva;

69. Research Tools [Internet]. 2011 Apr 18 [cited 2011 Apr 18]; Available from:http://www.wtgrantfdn.org/resources/overview/research_tools/research_tools
70. FAO Country Profiles - Cambodia. (2009). [cited April 15, 2012, ]; Available fromhttp://www.fao.org/countryprofiles/index.asp?iso3=KHM
71. Marriott, B., White, A., Hadden, L., Davies, J., & Wallingford, J. (2010). How well are infant and young child World Health Organization (WHO) feeding indicators associated with growth outcomes? An example from Cambodia - Maternal and Child Nutrition, 6(4), 358 – 373.
72. International Human Development Indicators - UNDP. (2011). [cited April 15, 2012]; Available from: http://hdrstats.undp.org/en/countries/profiles/KHM.html
73. WHO. (2005). Guiding principles for feeding non-breastfed children 6-24 months of age.Available from: http://whqlibdoc.who.int/publications/2005/9241593431.pdf
74. WHO (2012) Low birthweight newborns (percentage [cited April 24, 2012]; Available from: http://www.who.int/whosis/indicators/compendium/2008/2bwn/en/index.html
75. WHO. (2012). Complementary feeding. *Nutrition*. [cited April 24, 2012]; Available from:http://www.who.int/nutrition/topics/complementary_feeding/en/index.html
76. WHO. (2012). Promoting proper feeding for infants and young children. *Nutrition*. [cited April 24, 2012] Available from: http://www.who.int/nutrition/topics/infantfeeding/en/index.html
77. Research Tools [Internet]. 2011 Apr 18 [cited 2011 Apr 18];Available from: http://www.wtgrantfdn.org/resources/overview/research_tools/research_tools

## 10 Annexes

## 10.1 Logical Framework of the Research Project

| Project Objectives/Research Objective | Indicators | Means of Verification | Assumptions/Risks |
|---|---|---|---|
| **Project Impact:**<br>Child nutritional status is improved<br><br>**Research objective:**<br>Evaluate the impact of the project on child nutritional status in FAO food security projects | **Key Impact Indicators**:<br>a. Improved child nutritional status:<br>b. Reduced Stunting<br>c. Reduced Anemia<br>d. Reduced vit A deficiency | Baseline and Impact Surveys of children 0-23 months in FAO food security project and non-project areas | Households are willing to participate in all aspects of data collection |
| **Outcome:**<br>Complementary feeding (CF) practices and dietary intakes of children 0-23 months are improved<br><br>**Research objective:**<br>Assess changes in CF practices across interventionandcontrolhouseholds | a. Improved CF practices (Selected WHO IYCF)<br>b. Decreased prevalence of anemia, vit A deficiency<br>c. Increased diversity of foods fed to children 6-23 months<br>d. Increased frequency of feeding semisolid/solid foods<br>e. Mothers'sbehavior score | Onelevelofassessment: (Impact assessment) | The complementary feeding components are implemented according to plan and the TIPs correctly assesses the decision making processes at household level that lead to sustained changes in feeding practices |
| **Output 1:**<br>Knowledge and practices related to CF are Improved | Knowledge and practices (breastfeeding, CF and safe handling/preparation of food, active feeding, frequency of feeding, quantity, quality) of key change agents for child feeding practices are improved.<br><br>Decreased prevalence of ARI and diarrhoea. | Two levels of assessment (TIPs and Impact assessment) | The complementary feeding components are implemented according to plan and the TIPs<br>facilitators correctly assess the decision making processes at household level that lead to sustained changes in feeding practices |
| **Project activity: Training of TIPs facilitators** | TIPs facilitators are trained | | |
| **Research activities:**<br>TIPs consultants conducts the training of the TIPs facilitators according to the guidelines and | a. TIPs training is conducted according to the guideline.<br>b. TIPs facilitators are tested whether | TIPs training will be monitored and a pretest will be conducted and analyzed | Participants at the training are willing to be observed during the training and willing to |

| Project Objectives/Research Objective | Indicators | Means of Verification | Assumptions/Risks |
|---|---|---|---|
| ensures that TIPs facilitators have a common understanding of the food needs of family members most at risk of becoming malnourished and are familiarized on the approach to use when counseling the TIPs families. | they are familiar with nutrition education messages.<br>c. TIPs facilitators' capability conducting data assessment according to the TIPs guidelines is tested | using the TIPs toolkit for TIPs implementation. | undergo a final knowledge assessment.<br><br>TIPs Training has not been conducted in the time of the research. |
| **Project activity: TIPs implementation** | Behavior change messages and improved CF recipes developed and tested with mothers | | |
| Research activities:<br>TIPs formative research generates appropriate behavior change messages and CF recipes that are:<br>Seasonally appropriate,<br>nutritionally adequate,<br>culturally acceptable<br>affordable. | a. # of appropriate behavior change messages developed<br>b. Assessment of the appropriateness, feasibility and acceptability of nutrition education messages<br>c. Assessment of the cultural acceptability of recipes<br>d. Assessment of the affordability of recipes<br>e. Assessment of factors affecting feeding practices and nutritional status<br>f. Assessment of facilitators/nutrition promoters' knowledge and skills in dietary counseling (in terms of accuracy of content and quality of the interaction)<br>g. Assessment of facilitators/nutrition promoters' knowledge and skills in facilitating cooking demonstrations | Research team monitoring TIPs<br><br>In-depth qualitative and quantitative information of TIPs and control HH<br><br>Cost effectiveness verified and fine tuned through Linear Programming tool.<br><br>Qualitative research (focus groups) on message acceptability, cultural acceptability of recipes and affordability of new recipes | TIPs has not been conducted in the time of the research and no national IYCF guidelines are available<br><br>Partnerships with FAO food security projects are established, funded and initiated at the same time as the research |
| **Research activities**<br>The potential for meeting the nutritional | a. Assessment of the ability of local foods to meet nutrient requirements | Seasonal calendars of food availability developed | Food composition data are available for local foods (local |

| Project Objectives/Research Objective | Indicators | Means of Verification | Assumptions/Risks |
|---|---|---|---|
| requirements of children 6-23 months of age with locally available and affordable foods is assessed. | by season<br>b. % of nutrient requirements met by improved recipes<br>c. Calculation of energy and nutrient density of CF<br>d. Number of CF recipes that meet nutritional requirements by season<br>e. Assessment of the cost and affordability of improved local recipes | Locally appropriate recipes developed<br><br>Nutrient content of CF recipes, including energy and micronutrient density assessed | food varieties) or samples of local foods or recipes are collected for laboratory analysis |
| **Research activities**<br>Improvements in dietary intakes and child nutritional status | a. Assessment of dietary intakes<br>b. Assessment of child growth<br>c. Assessment of motormilestones<br>d. Biochemical assessment of micronutrient and morbidity status | Initial and follow-up assessments of diet, growth, motormilestones and collection of blood samples | No drop out or refusal to participate |
| **Project activity –dissemination of feeding recommendations and recipes** | Disseminate through local community groups messages and recipes developed through TIPs | | |
| **Research Activities**<br>Evaluate the effectiveness of disseminating behavior change messages and improved recipes for CF, in association with a food security intervention | a. Assess the process through which messages and recipes are promoted and taken up<br>b. Assess the factors that encourage or hinder uptake of recipes (strength of community organization, combination of FS interventions and CF interventions)<br>c. Assess the frequency and quality of nutrition education and dietary counseling with families and community groups<br>d. Assess the effectiveness of different delivery channels | Qualitative research (i.e. social mapping, focus groups discussion)<br><br>Survey assessments | Presence in communities of women's groups. Messages are delivered to all persons within family/community that influence behaviors and practices |

# 10.2 IMCF Cambodia workplan

| | 2012 | | | | | | | | | | | | 2013 | | | | | | | | | | | | 2014 | | | | | | | | | | | |
|---|---|---|---|---|---|---|---|---|---|---|---|---|---|---|---|---|---|---|---|---|---|---|---|---|---|---|---|---|---|---|---|---|---|---|---|---|
| | 1 | 2 | 3 | 4 | 5 | 6 | 7 | 8 | 9 | 10 | 11 | 12 | 1 | 2 | 3 | 4 | 5 | 6 | 7 | 8 | 9 | 10 | 11 | 12 | 1 | 2 | 3 | 4 | 5 | 6 | 7 | 8 | 9 | 10 | 11 | 12 |
| Literature Review | | | X | X | X | X | X | | | | | | | | | | | | | | | | | | | | | | | | | | | | | |
| Refindresearch design | | | X | X | X | X | X | | | | | | | | | | | | | | | | | | | | | | | | | | | | | |
| Baseline Survey I | | | | | | | | X | X | | | | | | | | | | | | | | | | | | | | | | | | | | | |
| Baseline I analysis&report | | | | | | | | X | X | X | | | | | | | | | | | | | | | | | | | | | | | | | | |
| Midtermsurvey | | | | | | | | | | | | | | | | X | | | | | | | | | | | | | | | | | | | | |
| Midterm survey results analysis and | | | | | | | | | | | | | | | | | X | X | X | X | | | | | | | | | | | | | | | | |
| Impact assessmentsurvey | | | | | | | | | | | | | | | | | | | | | | | | | | | | X | | | | | | | | |
| Impact assessment analysis and report | | | | | | | | | | | | | | | | | | | | | | | | | | | | | X | X | X | X | X | X | X | X |
| Workshop: discussionofresults | | | | | | | | | | | | | | | | | | | | | | | | | | | | | | | | | X | X | | |
| Analysis and research write-up and dissemination | | | | | | | | | | | | | | | | | | | | | | | | | | | | X | X | X | X | X | X | X | X | |
| Presentingdissertatio n | | | | | | | | | | | | | | | | | | | | | | | | | | | | | | | | | | | | X |

## 10.3 Participants Card

**Women 18-45 years:**

Hemoglobin (Hb, g/dl)

| | |
|---|---|
| < 8 | Severe anaemic |
| < 11 | Anaemic |
| 12 | Normal (non-pregnant) |
| 11 | Normal (pregnant) |

BMI

| | |
|---|---|
| < 18.5 | Underweight |
| 19 – 25 | Normal |
| 25 – 30 | Overweight |
| > 30 | Obese |

**Children under 2 years:**

| WAZ | HAZ | |
|---|---|---|
| 0 | 0 | Normal |
| <-2 | <-2 | Acute |
| <-3 | <-3 | Severe |

**Children 6-23 months:**

Hemoglobin (Hb, g/dl)

| | |
|---|---|
| < 7 | Severe anaemic |
| < 11 | Anaemic |
| ≥ | Normal |

### Participants Card

Name Caretaker: ______________________

Name Child: ______________________

participated in the Baseline Survey of IMCF joined MALIS – Justus Liebig University.

For further information please contact:

FAO Siem Reap Office
#575 Taneuy Street, Wat Bo Village
Salakamroeuk Commune, Siem Reap
Cambodia
Tel: +855 (0) 63 96 91 85

Interview

| | |
|---|---|
| Household and food security situation | ☐ |
| Child feeding practices | ☐ |

Clinical part

| | |
|---|---|
| Anthropometric measurements | ☐ |
| Test for edema (child) | ☐ |
| Assessment of motor milestones of child | ☐ |
| Hemoglobin prick test, mother | ☐ |
| Hemoglobin prick test, child | ☐ |
| Blood test for vit A & iron status, child | ☐ |

Results of anthropometry and hemoglobin

| | Height (cm) | Weight (kg) | BMI (kg/m²) | Hb (g/dl) |
|---|---|---|---|---|
| Mother | | | | |
| Father | | | | |

| Age of child (months) | Height (cm) | Weight (kg) | WAZ | HAZ |
|---|---|---|---|---|
| | | | | |

Edema: no ☐ yes ☐ Hb (g/dl) ☐

**Conclusion**

| | | | | |
|---|---|---|---|---|
| Nutritional status of mother alarming? | no | ☐ | yes | ☐ |
| Nutritional status of child alarming? | no | ☐ | yes | ☐ |

**→ If yes, please contact the next health post**

## 10.4 Intra-class correlation and minimal detectable effect size
(Reference 77)

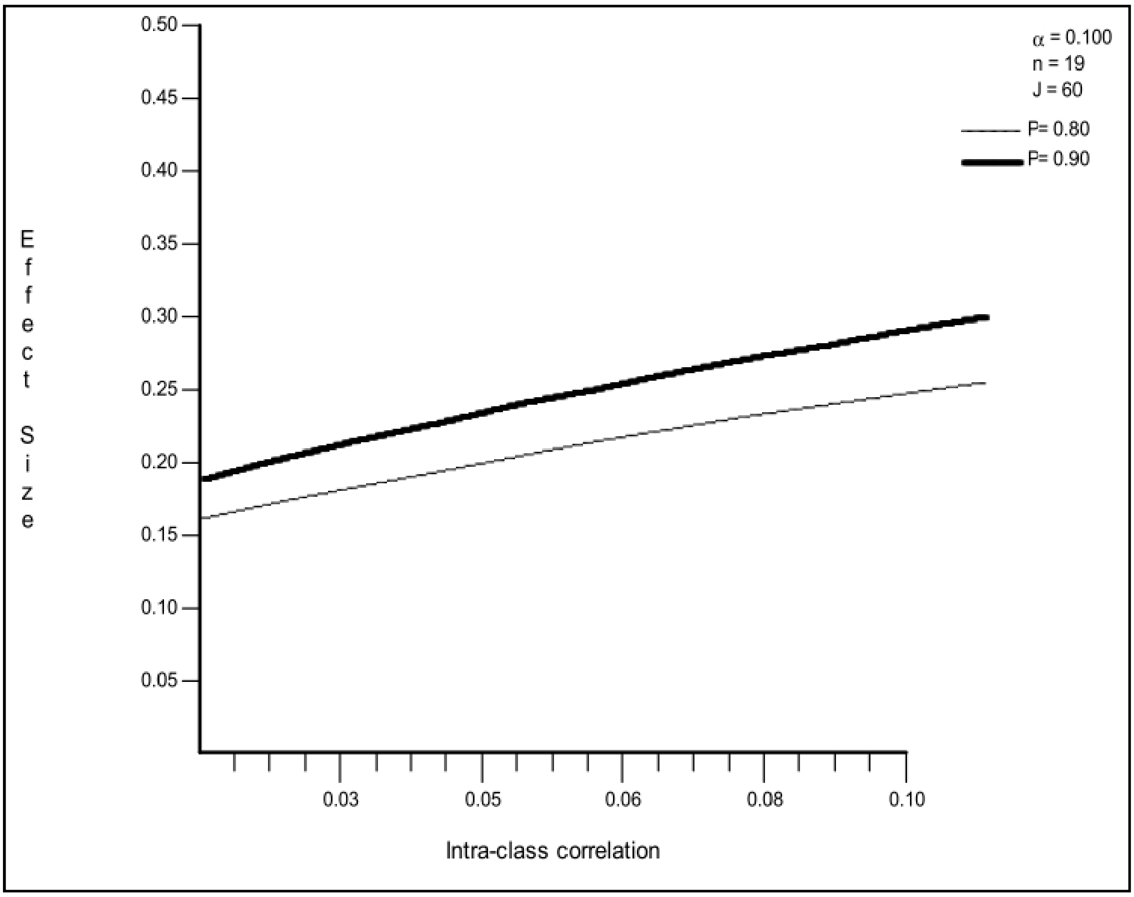


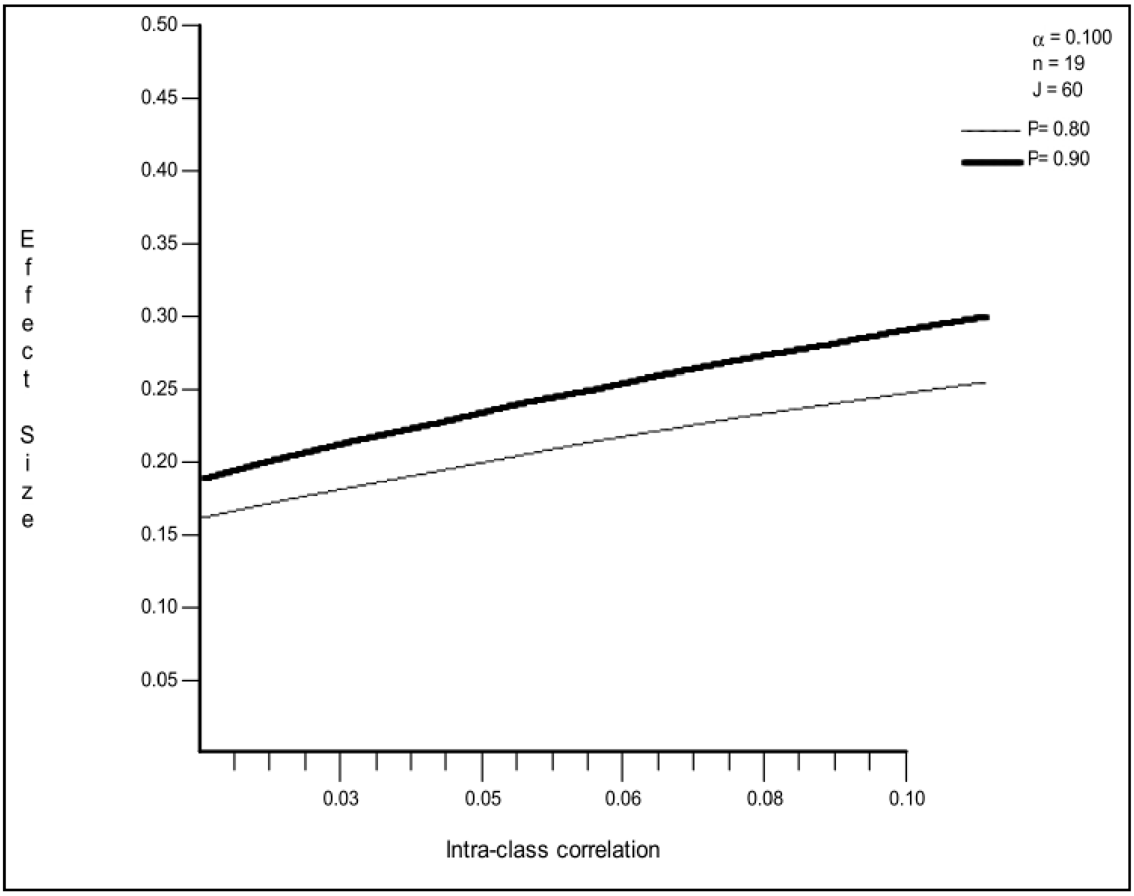

## 10.5 Budget IMCF Cambodia

| Component Description | year 1 | year 2 | year 3 | total |
|---|---|---|---|---|
| **Interviewers andtranslators** | 20.000,00 | 0,00 | 20.000,00 | |
| **Sub-total staff** | **20.000,00** | **0,00** | **20.000,00** | **40.000,00** |
| **Interviewer training** | 5.000,00 | 0,00 | 5.000,00 | |
| **Sub-total training** | **5.000,00** | **0,00** | **5.000,00** | **10.000,00** |
| **Localtransport (lumpsum)** | 5.000,00 | 2.500,00 | 5.000,00 | |
| **Sub-total travel** | **5.000,00** | **2.500,00** | **5.000,00** | **12.500,00** |
| **Expendableequipmentforsurveys** | 3.700,00 | 0,00 | 3.700,00 | |
| **SubtotalExpendable Equipment** | **3.700,00** | **0,00** | **3.700,00** | **7.400,00** |
| **Technical instruments** | | | | |
| **Office equipment** | | | | |
| **Subtotal Non Expendable Equipment** | | | | |
| **EthicalCommissionfee (US$)** | **400,00** | | | **400,00** |
| **Total US$** | | | | **70.300,00** |
